# TRAF4 hyperactivates HER2 signaling and contributes to Trastuzumab resistance in HER2-positive breast cancer

**DOI:** 10.1038/s41388-022-02415-6

**Published:** 2022-07-21

**Authors:** Yayun Gu, Huanyao Gao, Huan Zhang, August John, Xiujuan Zhu, Suganti Shivaram, Jia Yu, Richard M. Weinshilboum, Liewei Wang

**Affiliations:** Division of Clinical Pharmacology, Department of Molecular Pharmacology and Experimental Therapeutics, Mayo Clinic, Rochester, MN USA

**Keywords:** Breast cancer, Growth factor signalling

## Abstract

The HER2 receptor modulates downstream signaling by forming homodimers and heterodimers with other members of the HER family. For patients with HER2-positive breast cancer, Trastuzumab, an anti-HER2 monoclonal antibody as first-line therapy has shown significant survival benefits. However, the development of acquired resistance to Trastuzumab continues to be a significant obstacle. TNF receptor-associated factor 4 (TRAF4) upregulation was discovered to be associated with a worse clinical outcome. Here we identified TRAF4 overexpression as one of the putative mechanisms for HER2-positive breast cancer cells to maintain HER2 signaling during Trastuzumab treatment, while TRAF4 knockdown reduced HER2 stability and improved Trastuzumab sensitivity. Mechanistically, TRAF4 regulates HER2 level through its impact on SMAD specific E3 ubiquitin protein ligase protein 2 (SMURF2). The development of a membrane-associated protein complex containing HER2, TRAF4, and SMURF2 has been observed. SMURF2 bound to the HER2 cytoplasmic domain, and directly ubiquitinated it leading to HER2 degradation, whereas TRAF4 stabilized HER2 by degrading SMURF2 and inhibiting the binding of SMURF2 to HER2. Moreover, downregulation of TRAF4 has decreased the AKT/mTOR signaling. In conclusion, we discovered a new HER2 signaling regulation that involves the TRAF4-SMURF2 complex, a possible mechanism that might contribute to anti-HER2 resistance, making TRAF4 a viable target for treating HER2 + breast cancer.

## Introduction

The human epidermal growth factor receptor (HER) family receptor tyrosine kinases (EGFR/ErbB1, HER2/ErbB2, HER3/ErbB3, and HER4/ ErbB4) share a general structure that consists of an extracellular domain (ECD), a transmembrane domain (TMD), and an intracellular kinase encoding domain (ICD). The four members are functionally interdependent, and heterodimers can form between distinct HER proteins upon ligand binding to the ECD of one member, resulting in transphosphorylation of the ICDs and activation of a complex network of cell survival, proliferation, and mobility responses via regulating signals through a series of enzymes or adapters [1]. HER2 containing heterodimers are more hyperactive among the different HER dimers, since the ECD of HER2 is locked in a ligand-bound confirmation and the ICD of HER2 possesses strong kinase activity [1]. Furthermore, HER2 has been reported to enhance EGFR stability and activity as a binding partner [2]. Existing data suggest that HER2 features more promiscuous adapter binding sites comparing to HER3 and HER4 in terms of interactomes that can transduce signals to downstream pathways [3, 4].

While genomic alterations in HER genes have been documented in many human malignancies, HER2 gene amplification occurs in approximately 25–30% of all breast cancers [5]. Overexpression of HER2 allows for the constitutive activation of multiple downstream signaling molecules involved in tumor proliferation and survival, including mitogen-activated protein kinase (MAPK), phosphoinositide 3-kinase (PI3K/AKT), phospholipase C γ, protein kinase C (PKC) and signal transducer and activator of transcription (STAT), and thus serves as an oncogenic driver in breast cancer [1].

Trastuzumab (Herceptin) is a monoclonal antibody targeting HER2. Trastuzumab has been demonstrated to exert a variety of anti-tumor effects selectively in HER2-overexpressing tumor cells, including preventing dimerization and promoting endocytic degradation of HER2 receptor, and inducing cell cycle arrest and antibody-dependent cellular cytotoxicity. One-year Trastuzumab treatment is recommended for all HER2-positive breast cancer patients, but clinical benefits from extended treatment of Trastuzumab seem to be limited, partially due to the acquired resistance [6–9]. Common mechanisms for resistance include the abnormal activation of HER2 downstream signaling pathways, such as the AKT/mTOR pathway, and alterations that can occur in the apoptotic pathways [10–12]. However, additional potential resistant mechanism might be also due to the misregulation of the stability of the HER2, resulting in alteration in downstream signaling. Decoding the feedback mechanisms that breast cancer cells use to reverse the inhibited HER2 signaling is also critical for understanding the dynamic regulation of HER2 signaling network and would provide novel targets to overcome the resistance to HER2 inhibitors.

The tumor necrosis factor (TNF) receptor-associated factors (TRAFs) are signaling adapter proteins that couple signals from diverse cell membrane receptor families, such as the TNF receptors and the Toll-like/interleukin-1 receptors, with intracellular downstream signaling molecules. TRAFs 2–6 encode RING and zinc finger motifs that are essential for their adapter activities [13, 14]. The TRAF4 gene is located in the q11.2 region of chromosome 17, near the locus of HER2 oncogene. TRAF4 is an oncogenic adapter protein that has been shown to be overexpressed in roughly 20–30% of breast tumors. TRAF4 promotes polyubiquitination and degradation of its substrates [15, 16]. Membrane TRAF4 is commonly associated with critical biological processes such as cell polarity [17], cancer development, and metastasis [18]. It also contributes to drug resistance including chemo-resistance [19, 20]. As for the connection between TRAF4 and HER family, TRAF4 has been shown to directly bind to the juxtamembrane region of HER1/EGFR and increase kinase activation [21], whereas the potential interaction between TRAF4 and other HER receptors including HER2 remains unclear.

SMURF (SMAD specific E3 ubiquitin protein ligase protein, SMURF1 and SMURF2) is a C2-WW-HECT class E3 ubiquitin ligase that regulates cellular motility and polarity and was initially identified as a negative regulator of the TGF-beta signaling [22, 23]. SMURF and TRAF4 proteins exhibit a relatively uniform distribution across the cell membrane, cytoplasm, and nucleus, and their functions as adapters for different signaling pathways have been extensively studied [24, 25]. SMURF2, a significant partner for TRAF4, exhibits multifaceted functions serving as both tumor promoter and suppressor and is involved in apoptosis, metastasis, senescence, and genomic stability [22, 23, 26]. The TRAF4-SMURF2 complex has previously been demonstrated to counteractively regulate the stability of various proteins, including the IL-25R-inhibitory molecule DAZAP2 and TGF-β type I receptor (TβRI) [15, 27].

In this work, we discovered that TRAF4 and SMURF2 interacted with HER2 receptor within a protein complex and inversely regulated the stability of HER2. Specifically, our findings suggest that SMURF2 as an E3 ligase targeted HER2 for degradation, while TRAF4 shielded HER2 and its downstream signaling by degrading SMURF2 via its E3 ligase activity. Knockdown and knockout of TRAF4 dramatically reduced both the stability of HER2 receptor and the activation of HER2 downstream signaling pathways, improving sensitivity of breast cancer cells and PDX-derived organoids to Trastuzumab treatment.

## Results

### TRAF4 is upregulated and associated with poor prognosis in HER2+ breast cancer

**Fig. 1:**
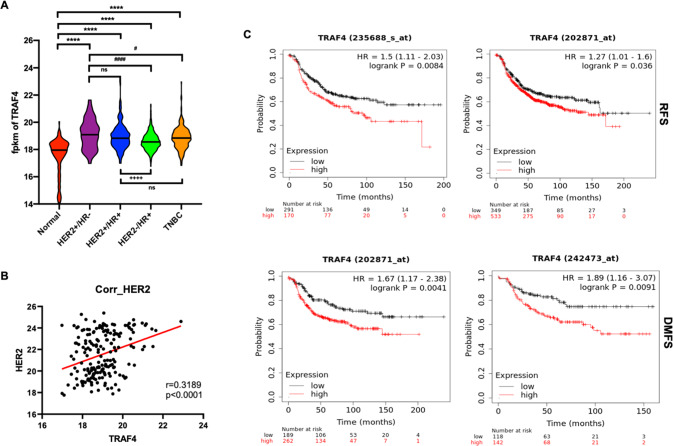
Upregulated TRAF4 is associated with poor prognosis in HER2+ breast cancer. **A** mRNA expression of TRAF4 in TCGA breast cancer cohort. HR (hormonal receptor positive, *n* = 661), HER2+/HR− (HER2 positive, hormonal receptor negative, *n* = 42), HER2+/HR+ (HER2 positive, hormonal receptor positive, *n* = 152), TN (triple negative, *n* = 243), and compared with normal (peripheral non-tumor tissue) (*n* = 113). ****p < 0.0001, ns not significant, ^#^*p* < 0.05, ^####^*p* < 0.0001, ^++++^*p* < 0.0001. **B** mRNA expression correlation between TRAF4 and HER2 in TCGA HER2 subtype sample set (*n* = 194). Pearson’s correlation coefficient (r) and statistical significance (*p* value) are shown as illustrated. **C** Kaplan–Meier analysis of HER2-positive patients comparing high TRAF4-expressing HER2+ tumors vs. low TRAF4-expressing tumors using KMplotter. (RFS relapse-free survival, DMFS distant metastasis-free survival).

Based on data extracted from the TCGA data set, we initially discovered a significant increase of TRAF4 mRNA level in breast cancer patient samples versus normal breast samples. While TRAF4 was overexpressed in almost all subtypes of breast cancer, the overexpression was more prominent in HER2+/HR− breast cancer samples compared to HER2−/HR+ and TN breast cancer samples (Fig. 1A). Moreover, TRAF4 and HER2 were shown to be co-expressed to some level in HER2+ breast cancer samples (*r* = 0.3189, *p* value < 0.0001) (Fig. 1B). We then evaluated the potential prognostic values of TRAF4 using various survival metrics, including relapse-free survival (RFS) and distant metastasis-free survival (DMFS). We found that higher TRAF4 expression in HER2+ tumors was significantly associated with poor prognosis (Fig. 1C). Taken together, these results implicated a potential role of the signaling adapter protein TRAF4 in the HER2-amplified subtype of breast cancer.

### TRAF4 inhibition improves Trastuzumab sensitivity in HER2+ breast cancer

**Fig. 2:**
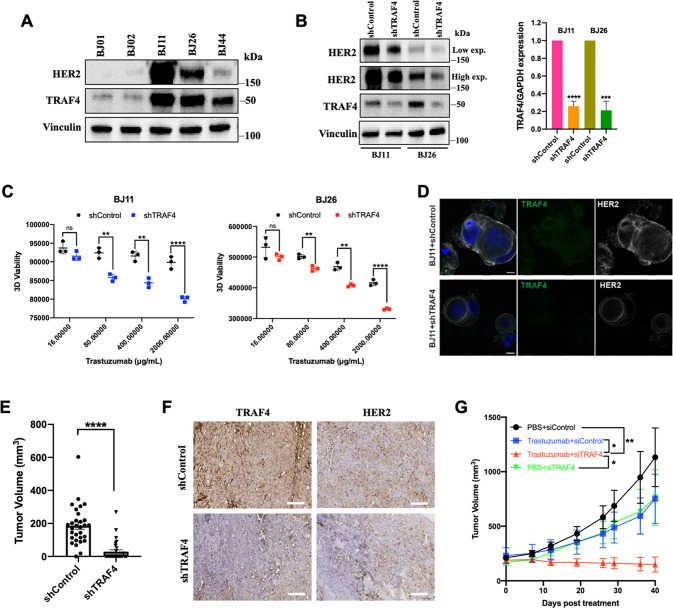
TRAF4 inhibition improves Trastuzumab sensitivity in HER2+ breast cancer. **A** Protein levels of TRAF4 and HER2 in tumor samples (BJ01, BJ02, BJ11, BJ26, BJ44) collected from breast cancer Patient-Derived Xenograft (PDX) models. **B** Effects of TRAF4 knockdown on HER2 protein expression in HER2+ breast cancer PDX-derived organoids generated from BJ11 and BJ26. Knockdown efficiency was verified at protein and mRNA levels by western blot and q-PCR analysis respectively. **C** 3D viability of HER2+ positive breast cancer PDX-derived organoids (BJ11 and BJ26) with or without TRAF4 knockdown. Organoids were transduced with shControl and shTRAF4 lentivirus followed by Trastuzumab treatment at the indicated concentrations for 3 days. **D** Immunofluorescence staining for TRAF4 and HER2 in HER2+ breast cancer PDX-derived organoid BJ11 with or without TRAF4 knockdown. Scale bar = 10 µm. **E** The sizes of shControl and shTRAF4 HCC1954 tumors were examined 30 days after implantation (*n* = 30). **F** Expression of TRAF4/HER2 in shControl and shTRAF4 HCC1954 tumors analyzed by Immunohistochemistry. **G** Weekly Trastuzumab and siTRAF4 therapy curves for shControl HCC1954 tumors, the tumor sizes of the four treatment groups were compared at day 40 post treatment (*n* = 7). ns not significant, *p < 0.05, **p < 0.01, ***p < 0.001, ****p < 0.0001.

Using the TCGA breast cancer cohorts, we found that HER2 and TRAF4 were amplified in 14% and 5% of all breast cancer respectively, and their amplifications were co-occurring in some cases (Supplementary Fig. S1). Our PDX tumor samples BJ01 and BJ02 were deriv from TN breast cancer, BJ11 and BJ26 were HER2+/HR− breast cancer, and BJ44 was HER2+/ER+ breast cancer. HER2 and TRAF4 expression levels varied among these tumor tissues. TRAF4 overexpression was observed in all three HER2+ tumors (BJ11, BJ26 and BJ44) compared to the TNBC tumors (BJ01 and BJ02) (Fig. 2A). Knockdown of TRAF4 by shRNA in HER2+/HR− PDX-derived organoids dramatically decreased HER2 protein levels (Fig. 2B). Moreover, knockdown of TRAF4 dramatically sensitized the organoids to Trastuzumab treatments compared to shRNA-controls (Fig. 2C). Expression of TRAF4/HER2 was detected in BJ11 organoids after TRAF4 knockdown (Fig. 2D). In an animal study using the HCC1954 xenograft model, we found that TRAF4 knockdown significantly reduced tumorgenicity of HCC1954 cells (Fig. 2E), whereas siTRAF4 intratumorally administration considerably downregulated HER2 expression and increased Trastuzumab sensitivity (Fig. 2F, G).

**Fig. 3:**
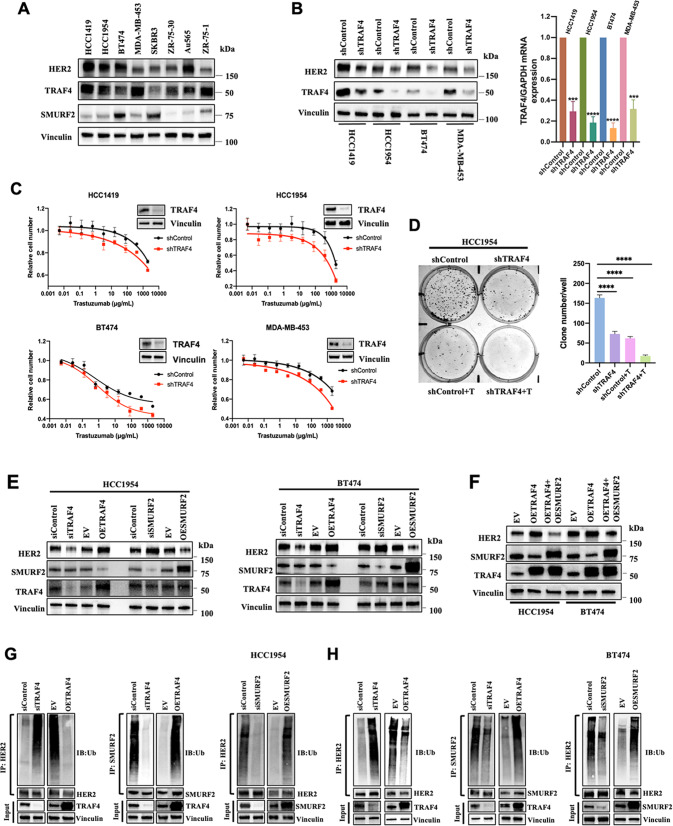
TRAF4 and SMURF2 inversely regulate HER2 stability. **A** Expression of HER2/TRAF4/SMURF2 in eight HER2+ breast cancer cell lines. **B** Effects of TRAF4 knockdown on HER2 protein expression in four HER2+ breast cancer cell lines. **C** Effects of TRAF4 knockdown on the sensitivity of HER2+ breast cancer cell lines to Trastuzumab treatment. **D** Quantification of colony formation in HCC1954 cells following transduction with shControl or shTRAF4 lentiviruses with or without 400 μg/mL Trastuzumab. **E** Effects of TRAF4/SMURF2 knockdown or overexpression on HER2 protein expression in HCC1954 and BT474 cells. **F** Effects of TRAF4 overexpression or TRAF4/SMURF2 co-overexpression on HER2 protein level in HCC1954 and BT474. **G**, **H** Effects of TRAF4/SMURF2 knockdown or overexpression on HER2/SMURF2 ubiquitination in HCC1954 and BT474 cells. Cells were treated with MG132 (20 μM) for 5 h before ubiquitination analysis.

We also determined HER2 and TRAF4 expression in eight breast cancer cell lines. Most cell lines showed an expected correlation between the two proteins. However, HER2 expression was low in the MDA-MB-453 and ZR-75-1 cell lines, despite high TRAF4 expression, indicating that other mechanisms regulating HER2/TRAF4 existed in some breast cancer cell lines (Fig. 3A). Furthermore, TRAF4 knockdown decreased HER2 expression in four HER2+ breast cancer cell lines (Fig. 3B) while enhancing Trastuzumab sensitivity (Fig. 3C). This result was further validated by colony formation analysis using HCC1954 cells. TRAF4 knockdown significantly reduced the number of HCC1954 cell colonies with or without Trastuzumab treatment (Fig. 3D). These findings suggested that TRAF4 potentially promotes HER2 stability, which leads to the resistance to the HER2 inhibitor, Trastuzumab. Since E3 ligases such as TRAF4 commonly induce target degradation rather than stabilize the target, we postulated that the process might involve another intermediate factor.

### TRAF4 and SMURF2 inversely regulate HER2 protein level

The UbiBrowser (ubibrowser.ncpsb.org) was subsequently used to search the E3 ubiquitination ligases that potentially ubiquitinate HER2 [28]. The results, presented in Supplementary Fig. S2, indicated that SMURF1 and SMURF2 might be the candidates as an intermediary protein linking TRAF4-HER2 together. Although a previous research has reported that SMURF1 can ubiquitinate HER2 when triggered by a compound JAC1 [29], SMURF1 targeted by TRAF4 has not been validated [24], whereas SMURF2 has been shown to be targeted by TRAF4 for promoting TGF-beta signaling and facilitating breast cancer metastasis [15]. After identifying the signaling adapter protein SMURF2 as a potential interactor, we proceeded to evaluate the TRAF4/SMURF2/HER2 connections.

To test our hypothesis that TRAF4 could stabilize HER2 via SMURF2, we first examined the expression levels of these protein in different HER2+ breast cancer cell lines and Fig. 3A shows that TRAF4 protein levels were shown to be inversely correlated with SMURF2 protein levels. We then used two cell lines, HCC1954 and BT474 to further study the relationship between these three proteins. In HCC1954 and BT474 cells, HER2 protein levels were downregulated following TRAF4 knockdown, and upregulated following induction of TRAF4 overexpression (Fig. 3E). In contrast, HER2 protein level was slightly increased when SMURF2 was knocked down and decreased after SMURF2 overexpression (Fig. 3E). These data implicated that TRAF4 and SMURF2 have opposing roles in the regulation of HER2 levels. To determine the relationship among the three proteins, we then performed sequential transfections with TRAF4 plasmids followed by SMURF2 plasmids in HCC1954 and BT474 cells. HER2 protein level increased after TRAF4 overexpression alone but was reduced when TRAF4 and SMURF2 were co-overexpressed, further supporting that TRAF4 regulates HER2 stability via SMURF2 (Fig. 3F).

Further ubiquitination analysis showed that in HCC1954 and BT474 cells, HER2 and SMURF2 degradation following TRAF4/SMURF2 knockdown or overexpression was ubiquitin-dependent (Fig. 3G, H), which is consistent with the E3 ligase activity of TRAF4/SMURF2. In addition, Trastuzumab treatment reduced the expression of SMURF2 and HER2, and increased the expression of TRAF4 in HCC1954 and BT474 cells in a dose-dependent manner (Supplementary Fig. S3A). In BT474 cells, increased ubiquitination of HER2 was detected under drug treatment, and the upregulation of TRAF4 was observed as early as 2 h after high concentration of Trastuzumab treatment (Supplementary Fig. S3B, C), implying that the elevated TRAF4 expression could be a feedback mechanism after cells exposed to Trastuzumab.

### TRAF4-SMURF2 mediates ubiquitination of HER2 via the proteasomal dependent pathway

**Fig. 4:**
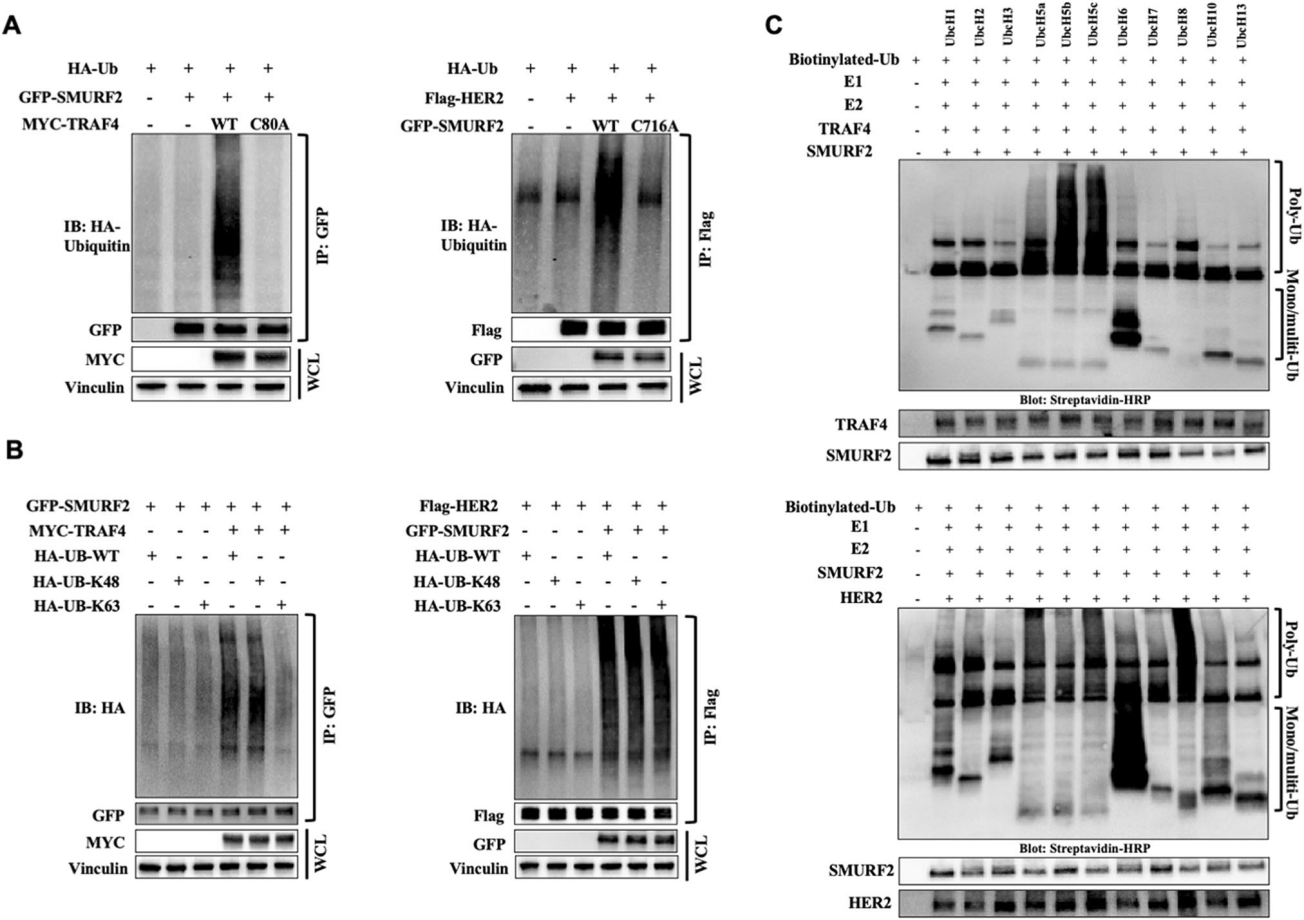
SMURF2 targets HER2 for ubiquitination dependent degradation, while TRAF4 targets SMURF2 for degradation. **A** Immunoprecipitation and ubiquitination analyses of TRAF4/SMURF2 and SMURF2/HER2 complexes in whole-cell lysates of ubiquitin expressing HCC1954 cells transfected with the indicated plasmids. Cells were treated with MG132 (20 μM) for 5 h before analysis. Whole-cell lysate (WCL) was loaded as an input. **B** Immunoprecipitation and ubiquitination analyses in the presence of WT or mutant (K48 and K63) ubiquitin in HCC1954 cells transfected with the indicated plasmids. Ubiquitinated SMURF2 and HER2 were detected following immunoprecipitation and immunoblotting. **C** In vitro ubiquitination analysis. Purified proteins, TRAF4, SMURF2 and HER2, E1, E2, and ubiquitin were mixed, incubated and subjected to immunoprecipitation followed by immunoblotting using indicated antibodies.

Given the ubiquitination activities of TRAF4 and SMURF2, we sought to test the roles of TRAF4 or SMURF2 as an E3 ligase within the protein complex. As demonstrated in Fig. 4A and Supplementary Fig. S4A, in both HCC1954 and HEK293T cells, the wild-type TRAF4 enhanced SMURF2 polyubiquitination, whereas the TRAF4 C80A mutant with abolished E3 ligase activity as described previously [15, 26, 27, 30], failed to elicit this effect. Although the wild-type SMURF2 polyubiquitinated HER2, no substantial ubiquitination of HER2 by SMURF2 C716A mutant was observed.

Furthermore, we sought to profile the specific lysine residues involved in ubiquitination. The most common ubiquitin linkage lysine 48 (K48) is generally associated with proteasomal protein breakdown, whereas the lysine 63 (K63) linkage usually results in the non-proteasomal post-translational modifications for modulating protein subcellular localization or interactions [31, 32]. We expressed the HA-tagged wild-type ubiquitin (HA-UB-WT), ubiquitin with only a lysine 48 residue (HA-UB-K48) and ubiquitin with only a lysine 63 residue (HA-UB-K63) in HEK293 cells and HCC1954 cells simultaneously expressing SMURF2 and TRAF4. Results suggested that the ubiquitination of SMURF2 by TRAF4 was mainly mediated through the K48 residue, while the polyubiquitination of HER2 by SMURF2 were both K48- and K63-dependent (Fig. 4B and Supplementary Fig. S4B). To explore the potential degradation pathways used for the ubiquitinated SMURF2/HER2, we blocked the proteasome route with MG132 and the lysosome pathway with Bafilomycin in the transfected HEK293T cells. As results shown in Supplementary Fig. S5, MG132 treatment, not Bafilomycin showed inhibitory effects on both TRAF4-mediated SMURF2 ubiquitination and SMURF2-mediated HER2 ubiquitination, suggesting that both TRAF4-mediated SMURF2 ubiquitination and SMURF2-mediated HER2 ubiquitination led to a proteasome-dependent degradation.

Further in vitro ubiquitination analyses were carried out using the purified proteins, ubiquitin E3 ligases or substrates, as well as the E1 activating enzymes, E2 (UbcHs) conjugating enzymes and biotinylated ubiquitin. The results demonstrated the ability for TRAF4 to polyubiquitinate SMURF2 in the presence of UbcH5b and UbcH5c (Fig. 4C upper panel). In the HER2 ubiquitination assays by SMURF2, polyubiquitination of HER2 was detected using UbcH5c and UbcH8, whereas mono/multi-ubiquitination of HER2 was detected in the reactions using UbcH6 (Fig. 4C lower panel). Altogether, these data support TRAF4 serving as an E3 ligase to protect HER2 by targeting SMURF2 for proteasome-dependent degradation within the protein complex TRAF4/SMURF2/HER2.

### TRAF4 and SMURF2 form a complex with HER2

**Fig. 5:**
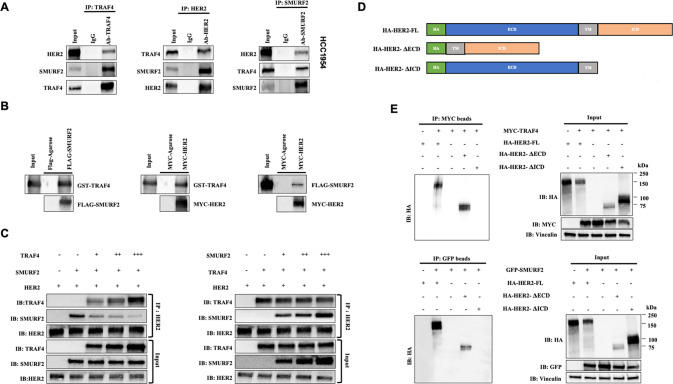
TRAF4/SMURF2/HER2 form a complex. **A** Endogenous interaction of TRAF4/SMURF2/HER2 detected by co-immunoprecipitation in HCC1954 cells. **B** In vitro binding assays of purified TRAF4, SMURF2 and HER2 proteins. **C** TRAF4 and SMURF2 compete for binding to HER2. Different amounts of pure TRAF4, SMURF2 and HER2 protein were combined, and HER2 was then pulled down for immunoblotting analyses with TRAF4 or SMURF2 antibodies. The symbols “−” and “+” indicate that 0 and 1 µg of protein is added, respectively. Symbols “++” and “+++” indicate the addition of 2 and 3 µg protein, respectively. **D** Diagram of HER2 protein truncations constructed (HA-HER2-FL: HA-HER2-full length, HA-HER2-ΔECD: extracellular domain deleted truncate, HA-HER2-ΔICD: complete intracellular domain deleted truncate). **E** Co-immunoprecipitation of TRAF4 /SMURF2 with HER2 full protein or truncated variants.

Following the discovery of functional interdependence between TRAF4, SMURF2 and HER2, we subsequently evaluated the endogenous physical interaction among TRAF4/SMURF2/HER2 in HER2+ breast cancer cell lines. TRAF4/SMURF2/HER2 complex formation was verified by co-immunoprecipitation analysis in four HER2-positive breast cancer cell lines studied (Fig. 5A and Supplementary Fig. S6A) and in transiently transfected HCC1954 cells and HEK293T cells (Supplementary Fig. S6B). Meanwhile, we further examined these interactions between purified proteins in vitro and discovered that the GST-TRAF4 protein can be pulled down by the FLAG-SMURF2 protein, and that both GST-TRAF4 and FLAG-SMURF2 protein can be pulled down by the MYC-HER2 protein (Fig. 5B), supporting direction interactions of the three proteins in the TRAF4/SMURF2/HER2 complex.

To evaluate any competition between TRAF4 and SMURF2 in binding to HER2 in vitro, we discovered that increasing levels of TRAF4 reduced the interaction between HER2 and SMURF2, whereas the increased SMURF2 failed to reduce the interaction of HER2/TRAF4, implying that upregulated TRAF4 may inhibit the HER2 and SMURF2 interaction in addition to decreasing SMURF2 (Fig. 5C). Moreover, we assessed the subdomains of HER2 that might bind to TRAF4 or SMURF2 protein. HER2 truncates with deleted extracellular domain (ΔECD) or intracellular domain (ΔICD) were constructed (Fig. 5D) and transfected into HEK293T cells for interaction analyses. IP results revealed that TRAF4/SMURF2 both bound to the cytoplasmic domain of HER2 (Fig. 5E), since only the HER2 ECD truncation preserved the capacity to bind TRAF4/SMURF2.

We also did co-immunoprecipitation studies using separated membrane and cytoplasmic fractions from HCC1954 and BT474 cells, and results suggested that the TRAF4/SMURF2/HER2 complex formation could occur both in cytosol and on membrane with more significant on membrane since HER2 is majorly distributed on membrane (Supplementary Fig. S6C). Then, we performed an immunofluorescence assay to identify the subcellular localizations of TRAF4/SMURF2/HER2 in HER2+ breast cancer cells (Supplementary Fig. S7). HER2 was mainly located on cell membrane with enhanced signal at cell-cell junctions. TRAF4 was dispersed throughout the entire cell with elevated cytoplasm and cell membrane specific signals. SMURF2 was distributed homogeneously across the cell. Pearson product-moment correlation coefficients (PCCs) between the signals of the three proteins in analyzed cell lines were computed and presented in Supplementary Table 4, and the coefficient values indicated an excellent correlation between the distributions of these three proteins.

### TRAF4 KO/KD downregulated AKT/mTOR signaling in HER2+ breast cancer

**Fig. 6:**
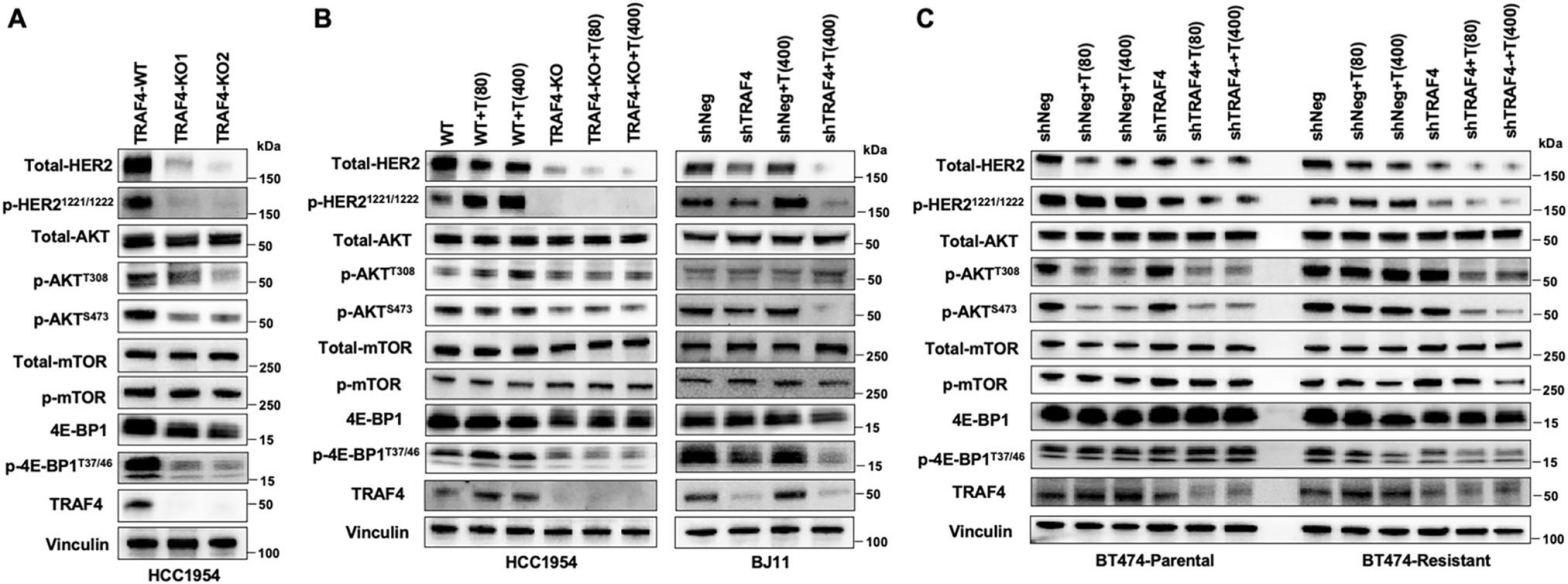
TRAF4 KO/KD inhibits activation of AKT/mTOR signaling pathway. **A** Detection of total proteins and phosphorylated proteins in the AKT/mTOR signaling cascade in TRAF4 knocked out (KO) HCC1954 cells. **B** Effects of Trastuzumab treatment (T: Trastuzumab, μg/mL) on the AKT/mTOR signaling activation in TRAF4 knocked out HCC1954 cells and in TRAF4 knockdown HER2+ breast cancer PDX-derived BJ11 organoids. **C** Effects of Trastuzumab treatment (T: Trastuzumab, μg/mL) on the AKT/mTOR signaling activation in TRAF4 knockdown (KD) parental and resistant BT474 cells. “Total” indicates total protein, “p” indicates phosphorylated form of protein.

The role of AKT/mTOR signaling in the development of Trastuzumab resistance has been elucidated [33, 34]. In this section, we set out to evaluate the effects of TRAF4 inhibition on activity of the HER2 downstream AKT/mTOR signaling pathway in HER2+ PDX BJ11 derived organoids and HER2+ breast cancer cells, including the Trastuzumab-resistant parental HCC1954 cells, the Trastuzumab-sensitive parental BT474 cells, and secondary Trastuzumab-resistant BT474 cells (sensitivity to Trastuzumab of these cells is shown in Fig. 3C and Supplementary Fig. S8 [11, 35–37]). We also established the CRISPR-mediated TRAF4 knockout (KO) HCC1954 cells and shRNA-mediated TRAF4 knockdown (KD) BT474 cells/BJ11 organoids. In HCC1954 and BT474 cells, TRAF4 KO/KD dramatically decreased the protein levels of both the total HER2 and phosphorylation form of HER2 (p-HER2^1221/1222^) (Fig. 6A–C). Strikingly, TRAF4 deletion effectively reduced the phosphorylation of AKT (p-AKT) and 4E-BP1 (p-4E-BP1^T37/46^) in HCC1954 cells and BJ11 organoids (Fig. 6A, B). These observations supported that TRAF4 effect on HER2 translates to its downstream functional impact.

Our data implies that the elevated TRAF4 after Trastuzumab treatment might be a molecular predictor of Trastuzumab resistance, which is generally accompanied by altered intracellular AKT/mTOR signaling. To test that, we compared the AKT/mTOR pathway activity in response to Trastuzumab in HCC1954-TRAF4 WT and KO cells. Trastuzumab marginally decreased the overall HER2 level in HCC1954-TRAF4 WT cells, while considerably increased the active p-HER2^1221/1222^ level, and slightly enhanced the phosphorylation of AKT at 308 sites (Fig. 6B), which reflects the status of Trastuzumab resistance as reported previously [38, 39]. By contrast, TRAF4 KO significantly reduced the levels of both total HER2 and phosphorylated HER2 in HCC1954 cells with or without Trastuzumab treatment. Interestingly, the addition of Trastuzumab did not significantly change the phosphorylation of most tested proteins, and only slightly increased the levels p-AKT ^T308^ and p-4E-BP1^T37/46^ in HCC1954 WT cells. While in the TRAF4 KO cells, Trastuzumab did not increase these phosphorylated protein levels compared with the nontreated TRAF4 KO cells (Fig. 6B). In addition, compared with WT cells treated with Trastuzumab, TRAF4 KO cells treated with Trastuzumab showed significantly reduced p-AKT ^T308/S473^ and p-4E-BP1^T37/46^ (Fig. 6B). To confirm this finding, we used a TRAF4 KD HER2+ PDX BJ11 derived organoids. Results showed that Trastuzumab increased HER2 phosphorylation in the BJ11-TRAF4 WT organoids, while TRAF4 KD reduced the expression of both total HER2 and p-HER2^1221/1222^, and also reduced the levels of p-AKT^S473^, and p-4E-BP1^T37/46^ (Fig. 6B).

Built on our discovery that TRAF4 regulates HER2 stability and its downstream signaling, and TRAF4 KO/KD could be used to overcome Trastuzumab resistance, we next investigated the combinatorial effects of Trastuzumab treatment and TRAF4 KD in parental and resistant HER2+ cells. As expected, sensitivity to Trastuzumab was found to be different between the parental and resistant BT474 cells (Supplementary Fig. S8A), and TRAF4 KD was effective in improving Trastuzumab sensitivity in both the parental and resistant BT474 cells (Supplementary Fig. S8B, C). Based on the phenotypes observed, we further studied the combinatorial impact on AKT/mTOR signaling within the parental and resistant BT474 cells (Fig. 6C). Trastuzumab decreased total HER2 but raised the p-HER2^1221/1222^ level in both cells, phosphorylation of AKT were only downregulated in the parental cells, while elevation of phosphorylated AKT in the resistant cells was consistent with prior findings [40]. Strikingly, in the TRAF4 KD cells, Trastuzumab treatment lowered both total HER2 and p-HER2^1221/1222^ levels. Both p-AKT^T308^ and p-AKT^S473^ were shown to be downregulated in parental and resistant cells. Overall, Trastuzumab treatment in TRAF4 KD BT474 resistant cells reduced AKT activity in a more promising way than that in parental cells (Fig. 6C).

## Discussion

In this study, TRAF4 and SMURF2, two classes of signaling adapter proteins, were found to bind simultaneously with the HER2 receptor at the intracellular domain and to inversely regulate HER2 stability and its downstream signaling via their E3 ligase activities, demonstrating the flexibility of the dynamic cellular signaling network in regulating the functions of HER2 by balancing the levels of their positive and negative regulators.

The TRAF4 protein is distributed widely across the whole cell from the plasma membrane to the nucleus and interacts with cell receptors (such as LTBR/TNFRSF3 and NGFR/TNFRSF16) on the membrane and with intracellular adapter proteins (including RPS6KB1, TGFB1I1, SMURF1, MAP3K4, NCF1, TICAM1, IRAK1 and TRAF6), executing a role in the activation of NF-kappa-B and JNK as well as regulating cell survival and apoptosis [41–43]. Its function as an oncogenic driver has been well characterized. We have added here that TRAF4 directly binds to and protects HER2 by degrading SMURF2, which also physically interacts with and degrades HER2. In addition, upregulated TRAF4 might also inhibit the interaction of SMURF2 with HER2 by competitively binding to HER2. Future studies will be required to determine how TRAF4 regulates SMURF2 and in turn HER2 in a more mechanistic fashion by characterizing the binding sites and by understanding whether this process might be also dependent on additional post-translational regulation. Together with the previously established role of TRAF4 in binding to the C terminal juxtamembrane region of HER1(EGFR) and participating in EGFR signaling activation [21], our findings further support the oncogenic roles of TRAF4 in breast cancer through increasing the HER2 signaling pathways. The possible interaction between TRAF4 and HER3/HER4 requires more investigation.

SMURF2 has been proposed as a tumor suppressor E3 ubiquitin ligase in normal cells, particularly through protecting cells against genome instability and carcinogenesis [44], whereas others have identified oncogenic activities for SMURF2 [45]. According to our results, SMURF2 directly binds to and decreases the stability of HER2, which is consistent with SMURF2’s tumor suppressive functions. However, SMURF2 might have implicated roles in regulating functions of all HER members, since it has been reported to bind, ubiquitinate, and protect EGFR from c-Cbl-mediated downregulation [46, 47]. Previously, the interaction between TRAF4 and SMURF2 has been reported, but the relationship between SMURF2 (previously identified as a negative regulator of TGF-beta receptor signaling) and other HER family proteins was unclear [22, 23, 48]. Here, we show that SMURF2 acts as an E3 ligase to inhibit HER2 receptor signaling. Interestingly, a recent study discovered that SMURF2 preferentially binds to mutant EGFR over wild-type EGFR and enhances the stability of altered EGFR [47], complicating its role in malignant cells. It is probable that SMURF2 is an adapter that is widely involved in the regulation of the entire HER family.

The four HER proteins (EGFR, HER2, HER3, HER4) form homodimers and heterodimers, with HER2 containing heterodimers being hyperactive. Trastuzumab and other HER2 targeting treatments have shown clinical success in the treatment of HER2+ breast cancer, however, efficacy is restricted by the emergence of drug resistance due to cellular feedback against the HER2 blockade [49–51]. We observed that TRAF4 upregulation is one strategy employed by HER2 + breast cancer cells to stabilize HER2 and desensitize Trastuzumab. Even though the treatment of Trastuzumab increased the degradation of HER2, at the same time, it also resulted in a rapid increase of TRAF4 protein levels, which translated to downstream AKT regulation. Therefore, TRAF4 KD/KO enhanced Trastuzumab sensitivity in resistant HER2+ breast cancer cells. TRAF4, we discovered, is important for maintaining the levels of several key components in the HER2 downstream pathway by safeguarding HER2 stability, as seen by the decreased total and phosphorylation HER2 level in TRAF4 KO/KD cells. Mechanisms of how Trastuzumab regulates TRAF4 expression and the correlation between TRAF4 levels and HER2 inhibitor sensitivity will be tested in future in vitro and in vivo studies.

**Fig. 7:**
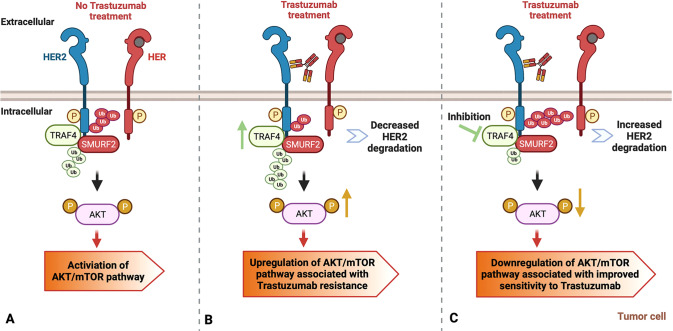
Schematic description of TRAF4/SMURF2-mediated regulation of HER2 signaling and Trastuzumab resistance in HER2+breast cancer cells. **A** TRAF4 and SMURF2 oppositely regulate HER2 and downstream AKT/mTOR pathway. SMURF2 binds to the HER2 cytoplasmic domain, and directly ubiquitinates it leading to HER2 degradation, whereas TRAF4 stabilizes HER2 by degrading SMURF2 and inhibiting the binding of SMURF2 to HER2. **B** TRAF4 upregulation mediates Trastuzumab resistance. Upregulated TRAF4 following Trastuzumab treatment protects HER2 receptor and downstream AKT/mTOR pathway via accelerating SMURF2 degradation and deportation from HER2. **C** TRAF4 inhibition leads to improved Trastuzumab sensitivity. Increased SMURF2 protein level after TRAF4 knockdown or knockout downregulates HER2 and downstream AKT/mTOR pathway.

To summarize, the model we present (Fig. 7) is that TRAF4 forms an E3 ligase complex with SMURF2, and this Ying-Yang TRAF4-SMURF2 regulatory complex regulates HER2 through balancing its stability and activity. Downregulated HER2 signaling by HER2 inhibitor promotes the onset of cellular feedback to upregulate HER2 signaling, in this case, through upregulation of TRAF4. Elevated TRAF4 levels lower the SMURF2 levels and possibly inhibit the interaction between HER2 and SMURF2, leading to restored HER2 stability and Trastuzumab resistance. On the contrary, when TRAF4 expression is suppressed, SMURF2 levels rise and HER2 degradation is accelerated, resulting in improved Trastuzumab sensitivity.

## Materials and methods

Please see Supplementary Methods for methodological descriptions of cell transfections, cell survival assay, immunoprecipitation, pull down assay, western blotting, immunofluorescence, immunohistochemistry, mRNA extraction, qRT-PCR analysis and animal study.

### Cell culture

The HER2+ breast cancer cell lines, HCC1954, BT474, HCC1419, Au565, ZR-75-30, and ZR-75-1 were maintained in Roswell Park Memorial Institute (RPMI) 1640 medium. The Human embryonic kidney HEK293T cells were cultured in Dulbecco’s modified Eagle medium (DMEM). SKBR3 cells were cultured in DMEM/F-12 (Dulbecco’s Modified Eagle Medium/Nutrient Mixture F-12) medium. MDA-MB-453 cells were cultured in Leibovitz’s L-15 Medium. All the medium contained 10% FBS (fetal bovine serum, Corning) and 1% penicillin/streptomycin (10,000 U/mL, Thermo Fisher). For establishment and maintenance of Trastuzumab-resistant cell, BT474 cells were grown in culture medium containing 15–20 µg/mL Trastuzumab for one year with media been renewed every 2–3 days. Resistance phenotype was determined by cell cytotoxicity assay.

### Patient-derived xenografts (PDXs) model

Breast cancer patient-derived xenografts (PDXs) were generated by implanting human breast tumor bulks subcutaneously to six to eight-week-old Cg-Prkdcscid Il2rgtm1Wjl/SzJ (NSG) female mice (The Jackson laboratory). Tumor samples were collected per the Breast Cancer Genome Guided Therapy Study (BEAUTY) (NCT02022202) as described in previous protocol [52]. All mice experiments were approved by Mayo Clinic Institutional Animal Care and Use Committee (IACUC) and were performed in compliance with Mayo Clinic IACUC guidelines.

### Organoid culture and 3D cell viability studies

Xenograft tumors were harvested for 3D organoids generation once tumors grew to 1.5 cm in diameter. First, fresh tumors were cut into pieces, digested by enzyme mix of Human Tumor Dissociation Kit (130-095-929, Miltenyi Biotech, Bergisch Gladbach, North Rhine-Westphalia, Germany) and incubated at 37 °C on a gentleMACS™ Octo Dissociator (130-095-937, Miltenyi Biotech) for 1 hour with gentle rotation. Then the digested mixture was filtered by a 70 μm MACS SmartStrainer to remove large debris, and then centrifuged at 300 × *g* for 10 min to pellet dissociated cells. Second, the cell pellet obtained was resuspended and treated with the Mouse Cell Depletion Purification Kit (130-104-694, Miltenyi Biotech) according to the manufacturer’s protocol to remove remaining mouse cells. The final purified breast cancer tumor cells were diluted to a final concentration of 2 × 10^4^ cells/mL and were then plated (100 µL/well) into 96-well ultralow attachment NanoCulture®Plates (NG-PLH9010, MBL life science, Tokyo, Japan) before being cultured at 37 °C to form 3D organoids. After 2 h incubation, control shRNA lentiviral particles or TRAF4 shRNA lentiviral particles (MOI = 3) were added to infect organoids in the presence of 5 μg/mL polybrene and cultured for 3 days followed by Trastuzumab treatment for additional 3 days at indicated concentrations. The susceptibility of the organoids to Trastuzumab was determined using the percentage of viability according to the manufacturer’s protocol of Promega 3D viability assay.

### CRISPR/Cas9-KO

For CRISPR/Cas9 mediated knockout of TRAF4 in HCC1954 cells, small guide RNAs (sgRNAs) were designed and cloned into the vector lentiCRISPRv2, and lentivirus was packaged with Lenti-X™ Packaging Single Shots (631275, TAKARA, Tokyo, Japan) in HEK293T cells. Transduced HCC1954 cells were selected with 2 μg/mL puromycin, and single colonies of cells were obtained by serial dilution. TRAF4 knockout clones were confirmed by immunoblotting with TRAF4 antibody.

### Site-directed mutagenesis and HER2 truncation construction

All HER2 truncate mutants were generated with the Q5 site-directed mutagenesis kit (New England Biolabs, E0554S). Primers used were listed in Supplementary Table 2.

### In vivo ubiquitination assay

HCC1954 cells/ BT474 cells were transiently transfected with TRAF4/SMURF2 targeting siRNAs or TRAF4/SMURF2 overexpressed plasmids. At 36 h post transfection, cells were replaced with fresh media containing 20 μM MG132 and cultured for 5 hours. Cells were then harvested, lysed, and subjected to immunoprecipitation assay. Ubiquitinated proteins were detected by western blot using anti-Ub antibody. Negative siRNA or empty vector were transfected for control.

### In vitro ubiquitination assay

MYC-TRAF4 or Flag-HER2 expressing plasmid was transiently transfected into HEK293T cells. Expressed protein was then purified using anti-MYC beads or anti-FLAG beads and eluted using 0.5 mg/mL Pierce c-Myc-Peptide (20170, Thermo Fisher Scientific) or 3X FLAG peptide (A36805, Thermo Fisher Scientific). In vitro ubiquitination assays were performed using ubiquitination kit (BML-UW9920-0001, Enzo Life Sciences, Farmingdale, New York, USA) following the manufacturer’s instructions. Briefly, the reaction was set up in a total of 25 μL volume with the following components: 2.5 μM biotinylated ubiquitin, 100 nM E1 enzyme, 1 μM E2 enzymes (UbcH1, UbcH2, UbcH3, UbcH5a, UbcH5b, UbcH5c, UbcH6, UbcH7, UbcH8, UbcH10, UbcH13/Mms2), 100 ng active human SMURF2 protein and purified TARF4 or HER2 protein. The mixture was incubated at 37 °C for 2 h and quenched by adding 2× non-reducing gel loading buffer before being analyzed by western blot analysis.

### Survival analysis

Survival analysis was performed with the TRAF4 mRNA levels (probe 235688_at/202871_at/242473_at) and relapse-free survival (RFS), distant metastasis-free survival (DMFS) using the kmplotter website (www.kmplot.com). TRAF4 expression cutoff was selected based on the recommendation from the kmploter software.

### Statistics

Statistical analyses were performed by using GraphPad Prism 8 software. Values were presented as mean ± SD. Experimental data were subjected to Student *t* test to calculate *p* value for the two-group comparisons with two tails. *p* < 0.05 was considered significant or as indicated in the figure legends. ns not significant, *p < 0.05, **p < 0.01, ***p < 0.001, ****p < 0.0001, ^++++^*p* < 0.0001. All data presented were the representative result of three independent repeated experiments.

## Supplementary information

Supplementary methods, figures, legends and tables
