# Supplementary material for: TRAF4 hyperactivates HER2 signaling and contributes to Trastuzumab resistance in HER2-positive breast cancer
Source: Oncogene. 2022 Jul 21;41(35):4119–29. doi: 10.1038/s41388-022-02415-6 (PMC9417995; doi:10.1038/s41388-022-02415-6)
Supplement: Supplementary file 1 — Supplementary methods, figures, legends and tables [file 41388_2022_2415_MOESM1_ESM.docx]

**SUPPLEMENTARY METHODS**

**Cell transductions and transfections**

All cells were cultured overnight to 70%-80% confluence in an incubator at 37 °C with 5% CO_2_ before transfections. The HER2+ breast cancer cells were transfected with shRNA plasmids using the Plasmid Transfection Reagent (sc-108061, Santa Cruz Biotechnology, CA, USA). Puromycin (2 μg/mL) was added to transfected cells to select stably transfected cells at 72 h post transfection. Lipofectamine 2000 (11668019, Thermo Fisher Scientific) was used to transfect plasmids and Lipofectamine RNAiMAX (13778500, Thermo Fisher Scientific) was used to transfect siRNA. Cells were then incubated and harvested at 48-72 h post transfection. Western blot analysis and qRT-PCR were performed to detect protein and mRNA expression levels for specific genes.

**Cell cytotoxicity assay**

Cells were seeded at 2000-4000 cells/well in 96-well plates and incubated overnight, followed by treatment with different concentration (2000, 400, 80, 16, 3.2, 0.64, 0.128, 0.0256 μg/mL) of Trastuzumab for 3 days. Then 10 μL CyQUANT reagent mix (C35012, Thermo Fisher Scientific) per well was added to the cells and incubated at 37 °C with 5% CO_2_. After a 1h incubation, the plates were read at Ex/Em (485 nm/530 nm) using an Infinite® M1000 microplate reader (Tecan, Männedorf, Switzerland) and cell proliferation was calculated based on the fluorescence intensity.

**Colony formation assay**

Cells (wild type and KO cells) were seeded into 6-well plates (500 cells/well) and cultured overnight, before being treated with Trastuzumab or control IgG for additional 7-12 days at 37 °C with 5% CO_2_. After treatment, the colonies were washed with PBS, fixed with ethanol for 30 min, and then stained with 0.5% w/v crystal violet for 2 h. The number of cell colonies was then counted with ImageJ software (NIH, Bethesda, Maryland, USA).

**Protein extraction**

Protein was extracted from cells or organoids using the NETN lysis buffer (100 mM NaCl, 20 mM pH 8.0 Tris-HCl, 0.5 mM EDTA, 0.5% (v/v) Nonidet P-40 (NP-40)) with plus 1x protease inhibitor (11836153001, Roche, Basel, Switzerland) and PhosSTOP (4906845001, Roche). To lyse the tumor, tumor in NETN buffer was homogenized on ice by an electronic homogenizer, supernatant was harvested by centrifugation at 12 000 × g for 10 min. Membrane protein and cytosolic protein were isolated using the Mem-PER™ plus membrane protein extraction kit (89842, Thermo Fisher Scientific) according to the manufacturer’s protocol. Protein concentrations were determined by Bradford assay.

**Immunoprecipitations**

Cell lysed using the Pierce™ IP Lysis Buffer (87787, Thermo Fisher Scientific) and centrifuged at 12 000 × g for 10 min at 4 °C. Equal amounts of total protein of cell lysates were incubated with 4 μg of antibody or control IgG overnight with a rotator at 4 °C. Then 30 μL of protein A/G magnetic beads (88802, Thermo Fisher Scientific) were added and the mixture were incubated at 4 °C for 4 h. The IP product was isolated by PureProteome magnetic stand (Millipore, Burlington, MA, USA) and washed three times by 800 μL lysis buffer. Proteins were dissolved in 2xSDS loading buffer and subjected to western blot analysis.

**Pulldown assay**

MYC-tagged HER2 protein, FLAG-tagged SMURF2 protein and GST-tagged TRAF4 protein were used to perform pulldown experiments, according to the instructions of the Pierce c-Myc-Tag IP/Co-IP kit (23620, Thermo Fisher Scientific) and FLAG Immunoprecipitation kit (FLAGIPT1, Sigma, St. Louis, MO, USA). Interactions between proteins were analyzed by western blotting.

**Western blotting**

Totally 20 μg of cell or tumor lysates were loaded in and separated by 4%-20% SDS-PAGE gels and transferred to PVDF membranes. The membranes were blocked with 5% milk in TBST, incubated with a primary antibody overnight at 4 °C, and then incubated with a secondary antibody for 1 h at room temperature. After antibody incubations, blots were developed with SuperSignal West Pico Chemiluminescent Substrate (34577, Thermo Fisher Scientific) and imaged using a ChemiDoc™ Touch Imaging System (BioRad, Hercules, CA, USA).

**Immunofluorescence**

Briefly, 1 x 10^5^ cells were plated in Nunc™ Glass Bottom Dishes (150680, Thermo Fisher Scientific) and cultured overnight to 70%-80% confluence. Cells were washed with PBS buffer before being fixed with 4% paraformaldehyde for 10 min at room temperature, followed by permeabilization using Tris-buffered saline (TBS) with 0.2% Triton X-100. Cells were then blocked in TBS buffer containing 0.2% Triton X-100/ 5% mouse serum/ 5% rabbit serum for 30 min, and then incubated with conjugated antibodies and DAPI overnight at 4 °C. For immunofluorescence staining of BJ11 organoids, the organoids were fixed with 4% paraformaldehyde for 10 min at room temperature, followed by permeabilization using 0.2% Triton X-100. Organoids were blocked in TBS buffer containing 0.2% Triton X-100/ 5% mouse serum/ 5% rabbit serum for 30 min and incubated with conjugated antibodies and DAPI overnight at 4 °C, then were transferred onto glass slides and sealed with coverslips. Fixed organoids were washed in PBS via centrifugation (200 ×*g* for 3 min at 4 °C). Conjugated antibodies were listed in Supplementary Table 1. Images were obtained using a Zeiss LSM780 Confocal Microscope system.

**Immunohistochemistry**

HCC1954 tumors were collected at day 40 post therapy and were fixed in 4% paraformaldehyde for 2 days, followed by dehydration with 30% sucrose in PBS for additional 2 days. Frozen sections (7µm in thickness) of the tumors from different groups were obtained and blocked with hydrogen peroxide blocking solution (ab64218, Abcam) for 10 minutes. Tumor slices were further blocked with 5% mouse serum/ 5% rabbit serum in PBS for 1 h, followed by 30 min incubation with primary antibodies listed in Supplementary Table 1 and another 30 min incubation with SiganlStain Boost IHC detection reagent HRP-mouse (#8125, Cell Signaling) for detecting mouse primary antibodies or SiganlStain Boost IHC detection reagent HRP-rabbit (#8114, Cell Signaling) for detecting rabbit IgGs. Slices were then sequentially stained with SiganlStain DAB Substrate (#8059, Cell Signaling) for 5 min and counterstained with Hematoxylin (#14166, Cell Siganling), before being dehydrated with ethanol and cleared with xylene. Slides were sealed with coverslips and scanned with a Zeiss Axio Scan. Z1 scanner.

**mRNA extraction and qRT-PCR**

Cells and organoids were maintained and collected as described above. Total RNA was extracted using Quick-RNA Miniprep kit (R1055, ZYMO RESEARCH, Irvine, CA, USA) following the manufacturer's manual. One hundred ng of total RNA was added for each Quantitative PCR analysis using Power SYBR™ Green RNA-to-CT™ 1-Step Kit (4391178, Thermo Fisher Scientific). Relative expression level of target proteins was calculated using the –ΔΔCt method and presented as mean ± SD from triplicate samples. Primers used are listed in Supplementary Table 2.

**Animal study**

HCC1954 tumors were created in six-week-old female athymic nude mice by implanting HCC1954 cells subcutaneously (5×10^6^ cells in 100µL PBS per mouse) (Foxn1^nu^, The Jackson laboratory). A total of 60 female nude mice were separated into two groups: one group of 30 mice received HCC1954 cells transduced with shControl encoded lentivirus, while the other group of 30 mice received HCC1954 cells transduced with shTRAF4 encoded lentivirus. Tumor volumes were calculated and compared between the two groups on day 30 after tumor implantation. Since day 30, the four groups of mice have been administered PBS+siControl, Trastuzumab+siControl, Trastuzumab+siTRAF4, and PBS+siTRAF4 on a weekly basis for 4 weeks. Trastuzumab was given intraperitoneally in PBS (20 mg/kg per dose), while siRNAs were incubated with Invivofectamine 3.0 Reagent (IVF3001, Invitrogen) according to the manufacturer's instructions before being injected intratumorally (2 µg siRNAs in 10 µL mix solution per tumor). On day 40 after therapy, tumors were collected. siRNAs used were listed in Supplementary Table 1 and Supplementary Table 3.

**SUPPLEMENTARY FIGURES**

**
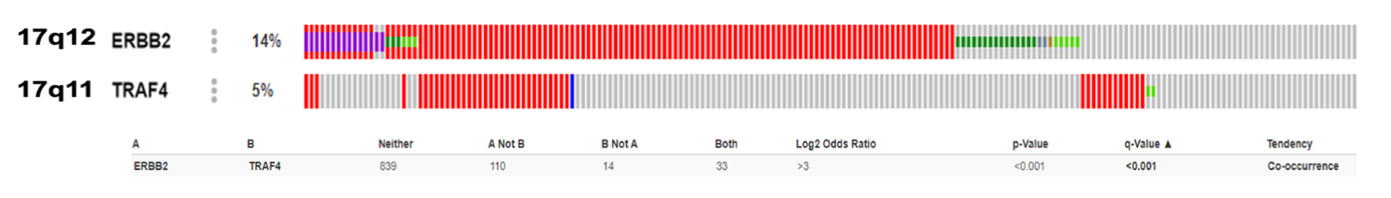
**

**Supplementary Figure 1. Co-occurrence analysis of HER2 and TRAF4 in human breast cancer. Data was subtracted from The Cancer Genome Atlas (TCGA) provisional dataset.**

**
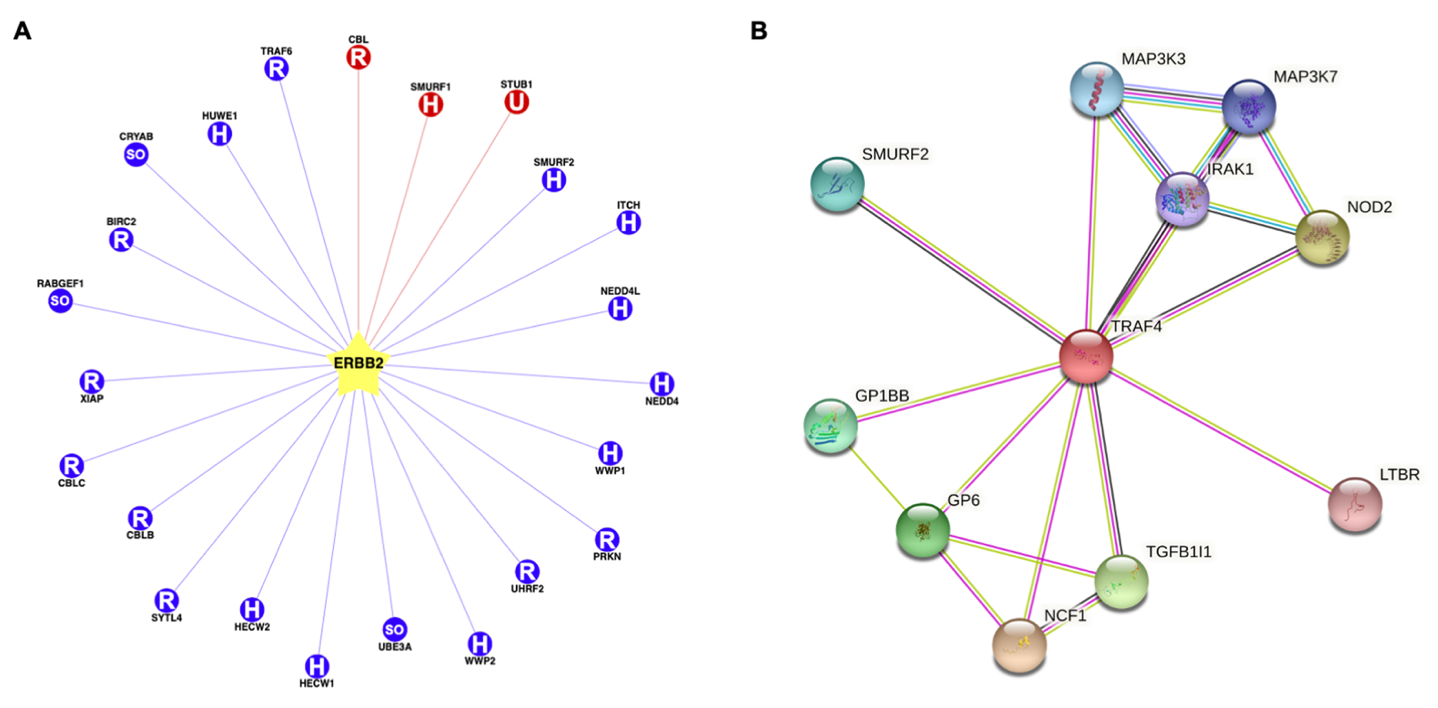
**

**Supplementary Figure 2. Prediction of HER2 and TRAF4 interacting factors.**

(A) The HER2 targeting E3 ubiquitin ligases were predicted using the database from ubibrowser.ncpsb.org. (B) Interaction map between TRAF4 and SMURF2 using the STRING database (string-db.org/)


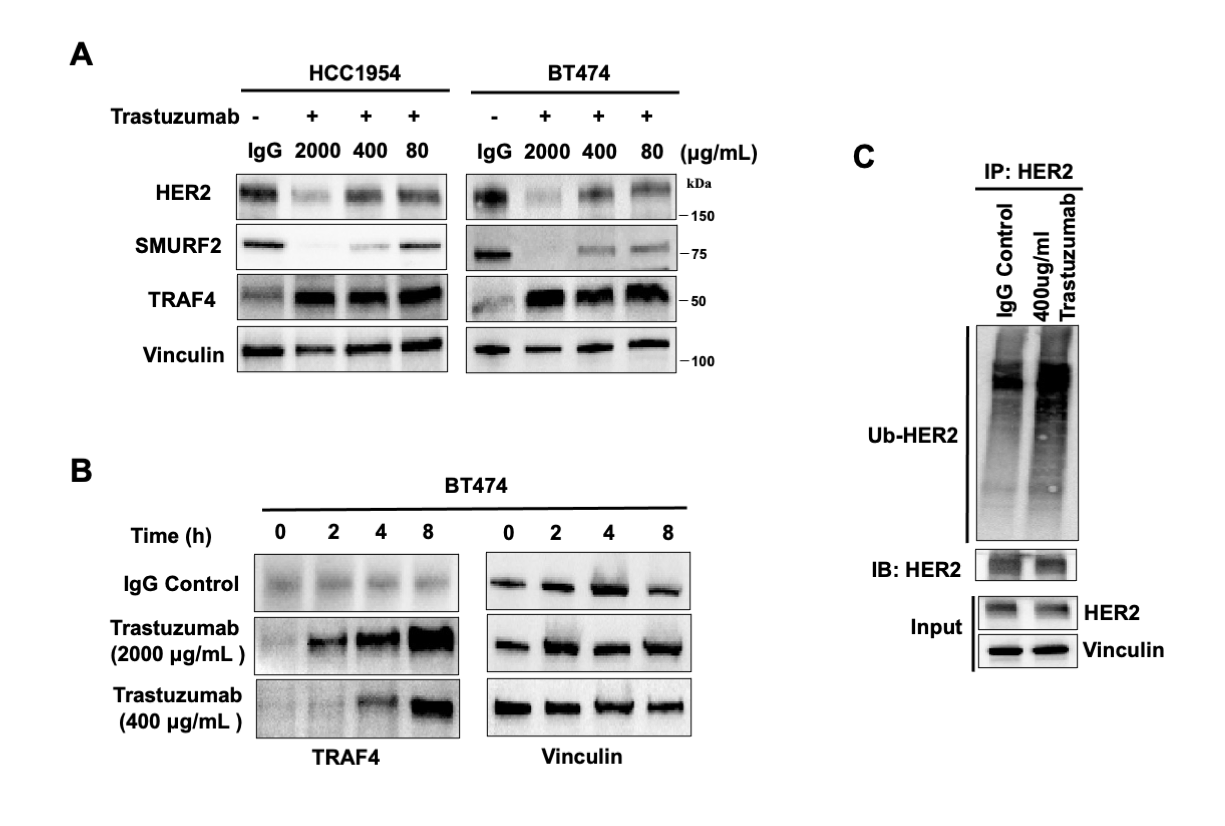


**Supplementary Figure 3. Protein levels of HER2, TRAF4 and SMURF2 in Trastuzumab-treated and non-treated HER2+ breast cancer cells.**

(A) TRAF4, SMURF2 and HER2 protein levels in BT474 and HCC1954 cells under different concentrations (2000 μg/mL, 400 μg/mL and 80 μg/mL) of Trastuzumab treatment. (B) TRAF4 protein levels in BT474 cells under Trastuzumab treatment detected from 0 to 8 h. (C) Ubiquitination of HER2 in BT474 cells post Trastuzumab treatment.


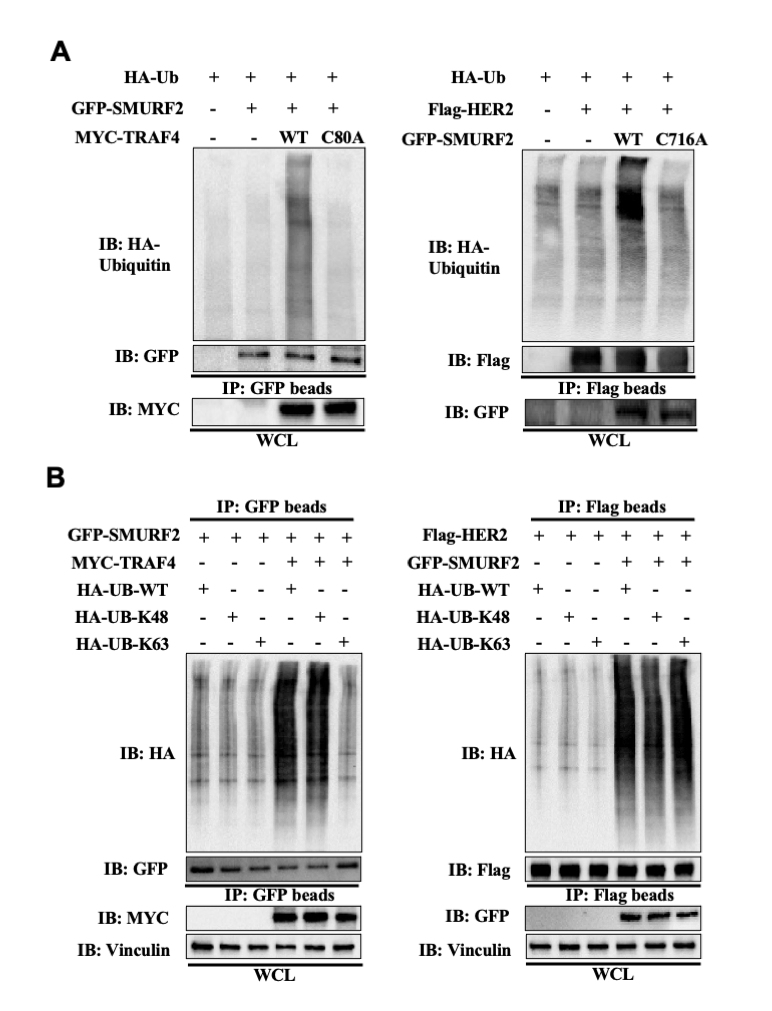


**Supplementary Figure 4. SMURF2 targets HER2 for ubiquitin-dependent degradation, whereas TRAF4 targets SMURF2.**

TRAF4/SMURF2 and SMURF2/HER2 complexes were immunoprecipitated and ubiquitinated in whole cell lysates (WCL) of ubiquitin-expressing HEK293T cells transfected with the corresponding plasmids. Before analysis, cells were treated with MG132 (20 μM) for 5 h. As an input, WCL was used. In HEK293T cells, immunoprecipitation and ubiquitination analyses were performed in the presence of WT or mutant (K48 and K63) ubiquitin. Following immunoprecipitation and immunoblotting, ubiquitinated SMURF2 and HER2 were discovered.


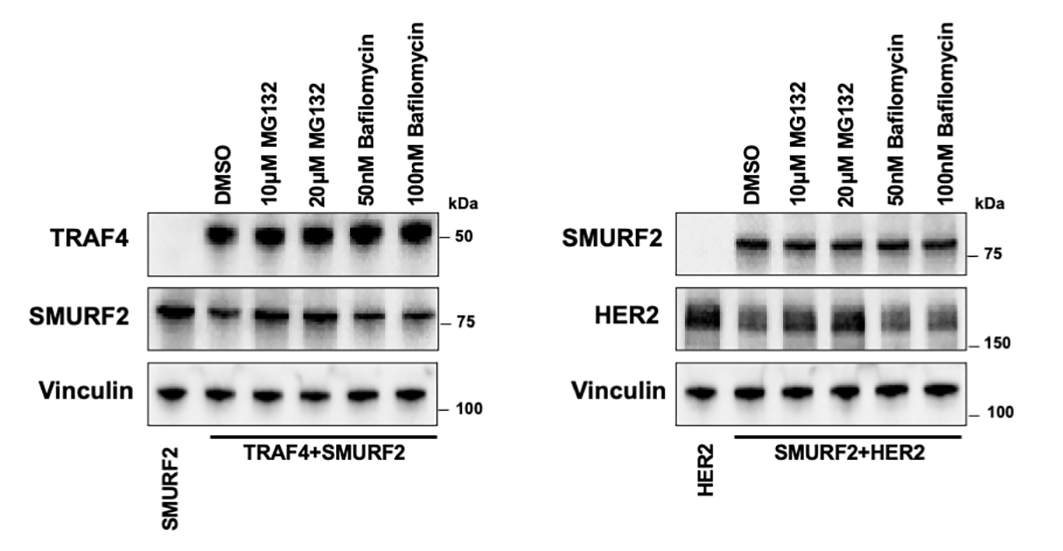


**Supplementary Figure 5. TRAF4 mediated degradation of SMURF2 and SMURF2 mediated degradation of HER2 are primarily proteasomal dependent.**

HEK293T cells were transfected with indicated plasmids for 48 h followed by DMSO, MG132 (10 μM/20 μM) or Bafilomycin (50 nM/100 nM) treatment for 5 hours.


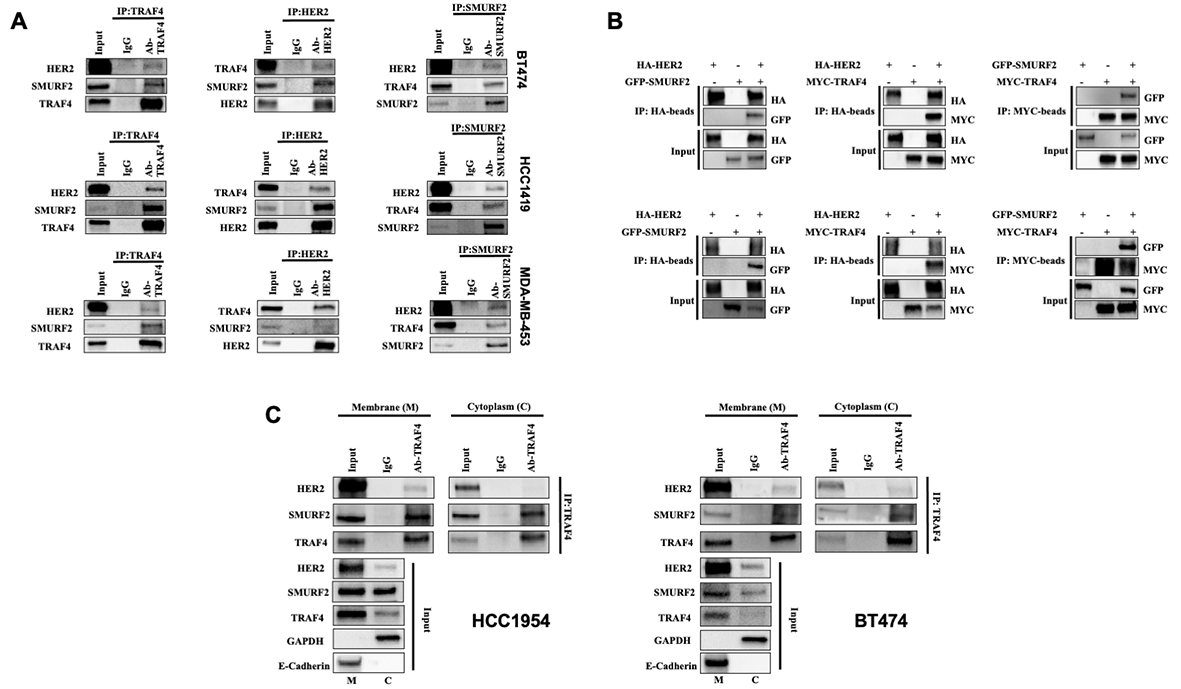


**Supplementary Figure 6. TRAF4, SMURF2 and HER2 form a complex in HER2+ breast cancer cells.**

(A) Endogenous interactions of TRAF4/SMURF2/HER2 in BT474/HCC1419/MDA-MB-453 cells. (B) Immunoprecipitation of TRAF4/SMURF2/HER2 interaction in transiently transfected HCC1954 (upper panel) and HEK293T (lower panel) cells. (C) Interactions of TRAF4 with SMURF2/HER2 in membrane fractions and cytoplasmic fractions isolated from HCC1954 and BT474 cells.

**
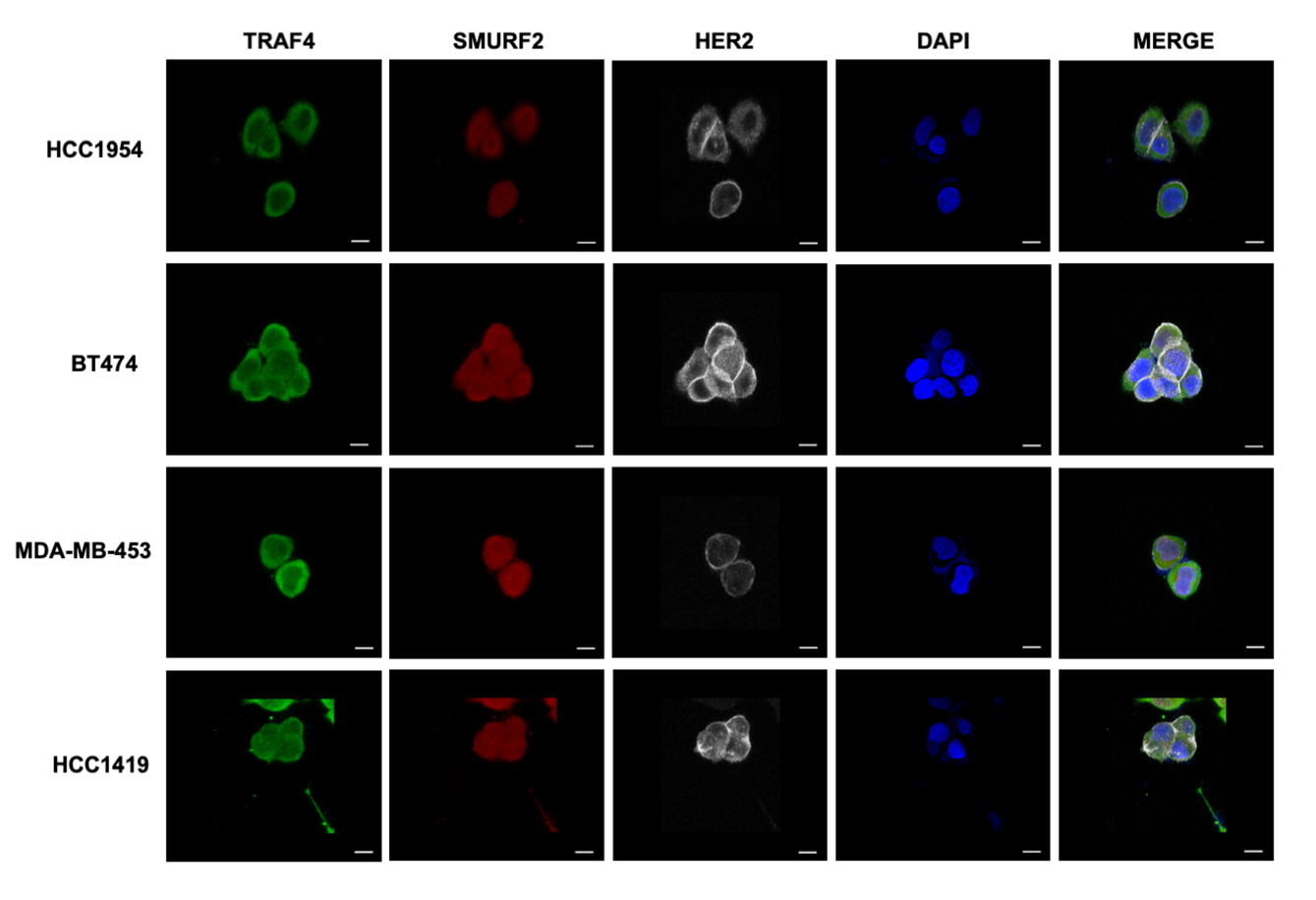
**

**Supplementary Figure 7. Subcellular localization of TRAF4, SMURF2 and HER2.**

Subcellular localization of TRAF4, SMURF2 and HER2 was detected by immunofluorescence staining in HCC1954/BT474/MDA-MB-453/HCC1419 cells.


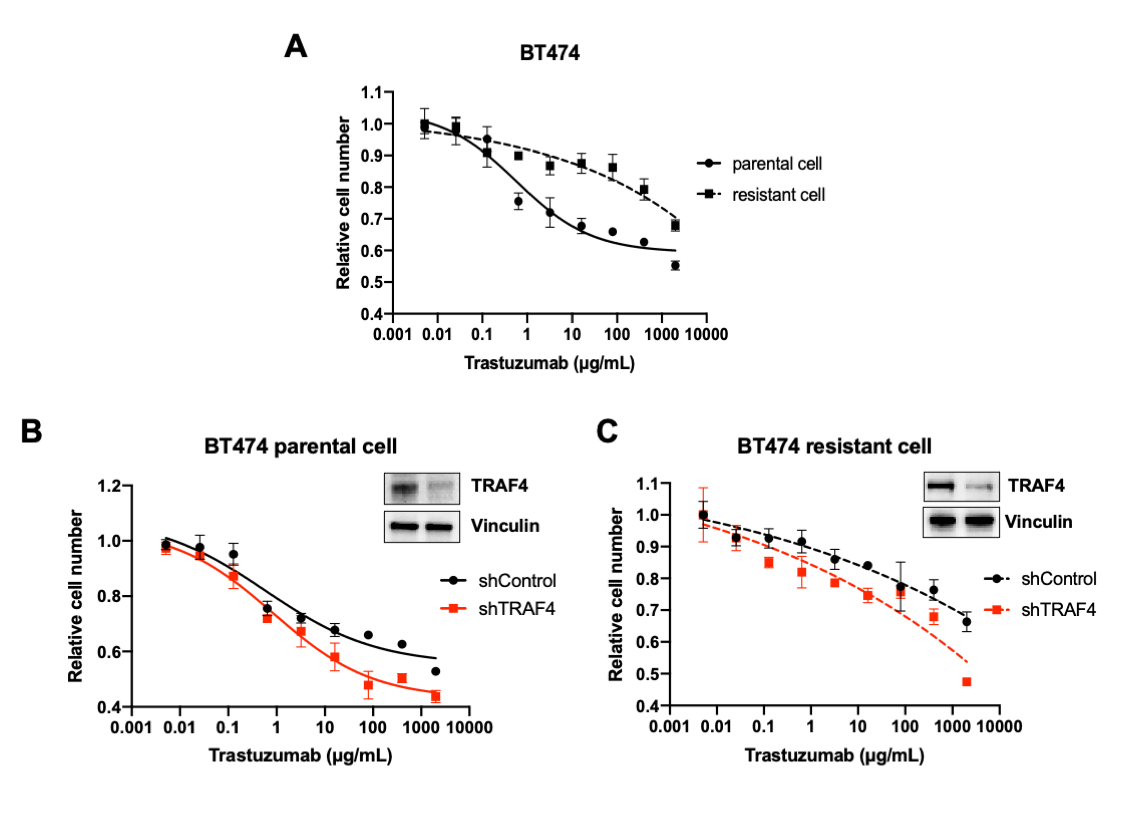


**Supplementary Figure 8. TRAF4 knockdown improves Trastuzumab sensitivity in parental and resistant BT474 cells.**

Relative cell number was measured using the CyQUANT assay in the BT474 cells. Cells transfected with TRAF4 shRNA (shTRAF4) or shControl vector were treated with different concentrations of Trastuzumab. Experiments were performed in triplicate, data were expressed as the mean ± SD.

**Supplemental Tables**

**Supplementary Table 1. List of reagents, antibodies, plasmids and proteins.**

| Products | Company | Catalog number |
| --- | --- | --- |
| Herceptin (trastuzumab) | Genentech, South San Francisco, CA, USA | 00010086840 |
| TRAF4 antibody | Abcam, Burlingame, CA, USA | ab245666 |
| TRAF4 antibody | PROTEINTECH NORTH AMERICA, Rosemont, IL, USA | 66755-1g |
| HER2 antibody | PROTEINTECH NORTH AMERICA, Rosemont, IL, USA | I8299-1-AP |
| SMURF2 antibody | Cell signaling Technology, Danvers, MA, USA | 120243 |
| SMURF2 antibody | Novus Biologicals,  [Littleton, CO](https://www.google.com/search?q=Littleton,+Colorado&stick=H4sIAAAAAAAAAOPgE-LSz9U3sEw3N48vU-IEsdMtcorTtIwyyq30k_NzclKTSzLz8_Tzi9IT8zKrEkGcYquM1MSUwtLEopLUomKFnPxksPAiVmGfzJKSnNSS_DwdBef8nPyixJT8HayMAKTSGuBmAAAA&sa=X&ved=2ahUKEwippNzxxvzzAhWzAp0JHcgpD-EQmxMoAXoECDEQAw), USA | NBP2-57554 |
| Ubiquitin antibody | Invitrogen, [Waltham, MA](https://www.google.com/search?q=Waltham&stick=H4sIAAAAAAAAAOPgE-LUz9U3MDNLKUxS4gAxM6qMTbW0spOt9POL0hPzMqsSSzLz81A4VhmpiSmFpYlFJalFxYtY2cMTc0oyEnN3sDICANGzN1FQAAAA&sa=X&ved=2ahUKEwjjxcjWxvzzAhXOGs0KHcZ2A2YQmxMoAXoECEgQAw), USA | 14-6078-82 |
| Normal rabbit IgG antibody | Cell signaling Technology, Danvers, MA, USA | 2729 |
| Anti-rabbit IgG, HRP-linked Antibody | Cell signaling Technology, Danvers, MA, USA | 7074 |
| Anti-mouse IgG, HRP-linked Antibody | Cell signaling Technology, Danvers, MA, USA | 7076 |
| AKT antibody | Cell signaling Technology, Danvers, MA, USA | 4685 |
| mTOR antibody | Cell signaling Technology, Danvers, MA, USA | 2983 |
| Vinculin antibody | Sigma, St. Louis, MO, USA | V9264 |
| Anti-MYC antibody | Cell signaling Technology, Danvers, MA, USA | 2276S |
| Anti-GFP antibody | Abcam, Burlingame, CA, USA | ab290 |
| Anti-GST antibody | Cell signaling Technology, Danvers, MA, USA | 2622 |
| Streptavidin-HRP antibody | Cell signaling Technology, Danvers, MA, USA | 3999 |
| Phospho-HER2/ErbB2 Antibody Sampler Kit | Cell signaling Technology, Danvers, MA, USA | 9923T |
| Phospho-HER2/ErbB2 (Tyr877) Antibody | Cell signaling Technology, Danvers, MA, USA | 2241T |
| Phospho-Akt Pathway Antibody Kit | Cell signaling Technology, Danvers, MA, USA | 9916T |
| mTOR Substrates Antibody Kit | Cell signaling Technology, Danvers, MA, USA | 9862T |
| 4E-BP1 (53H11) Rabbit mAb | Cell signaling Technology, Danvers, MA, USA | 9644T |
| SMURF2 Polyclonal Antibody, ALEXA FLUOR® 594 Conjugated | Bioss antibody, Woburn, MA, USA | bs-4056R-A594 |
| CoraLite®488-conjugated TRAF4 Monoclonal antibody | PROTEINTECH NORTH AMERICA, Rosemont, IL, USA | CL488-66755 |
| Alexa Fluor® 647 anti-human CD340 (erbB2/HER-2) | BioLegend, [San Diego, CA](https://www.google.com/search?q=San+Diego&stick=H4sIAAAAAAAAAOPgE-LSz9U3MCs3SSpKVuIAsc0Ny4q0jDLKrfST83NyUpNLMvPz9POL0hPzMqsSQZxiq4zUxJTC0sSiktSiYoWc_GSw8CJWzuDEPAWXzNT0_B2sjABuVANPWwAAAA&sa=X&sqi=2&ved=2ahUKEwiPiumFxfzzAhXxR_EDHYNqBVwQmxMoAXoECD0QAw), USA | 324412 |
| MYC-tagged TRAF4 plasmid | Origene, [Rockville, MD](https://www.google.com/search?q=Rockville,+Maryland&stick=H4sIAAAAAAAAAOPgE-LSz9U3qEpPMjUpV-IAsZMqkpK0tLKTrfTzi9IT8zKrEksy8_NQOFYZqYkphaWJRSWpRcWLWIWD8pOzyzJzclJ1FHwTiypzEvNSdrAyAgAR6wjwXQAAAA&sa=X&ved=2ahUKEwjD-NqaxfzzAhWRZM0KHTjhDWEQmxMoAXoECDcQAw), USA | RC200345 |
| GFP-tagged SMURF2 plasmid | Origene, [Rockville, MD](https://www.google.com/search?q=Rockville,+Maryland&stick=H4sIAAAAAAAAAOPgE-LSz9U3qEpPMjUpV-IAsZMqkpK0tLKTrfTzi9IT8zKrEksy8_NQOFYZqYkphaWJRSWpRcWLWIWD8pOzyzJzclJ1FHwTiypzEvNSdrAyAgAR6wjwXQAAAA&sa=X&ved=2ahUKEwjD-NqaxfzzAhWRZM0KHTjhDWEQmxMoAXoECDcQAw), USA | RG210866 |
| pCMV6-Entry negative control vector | Origene, [Rockville, MD](https://www.google.com/search?q=Rockville,+Maryland&stick=H4sIAAAAAAAAAOPgE-LSz9U3qEpPMjUpV-IAsZMqkpK0tLKTrfTzi9IT8zKrEksy8_NQOFYZqYkphaWJRSWpRcWLWIWD8pOzyzJzclJ1FHwTiypzEvNSdrAyAgAR6wjwXQAAAA&sa=X&ved=2ahUKEwjD-NqaxfzzAhWRZM0KHTjhDWEQmxMoAXoECDcQAw), USA | PS100001 |
| pCMV6-AC-GFP negative control vector | Origene, [Rockville, MD](https://www.google.com/search?q=Rockville,+Maryland&stick=H4sIAAAAAAAAAOPgE-LSz9U3qEpPMjUpV-IAsZMqkpK0tLKTrfTzi9IT8zKrEksy8_NQOFYZqYkphaWJRSWpRcWLWIWD8pOzyzJzclJ1FHwTiypzEvNSdrAyAgAR6wjwXQAAAA&sa=X&ved=2ahUKEwjD-NqaxfzzAhWRZM0KHTjhDWEQmxMoAXoECDcQAw), USA | PS100010 |
| HA-tagged HER2 plasmid | Sino Biological US Inc.Wayne, PA, USA | HG10004-NY |
| FLAG-tagged HER2 plasmid | Sino Biological US Inc.Wayne, PA, USA | HG10004-CF |
| pCMV3-SP-N-HA negative control vector | Sino Biological US Inc.Wayne, PA, USA | CV021 |
| pCMV3-C-FLAG negative control vector | Sino Biological US Inc.Wayne, PA, USA | CV012 |
| Control shRNA Lentiviral Particles-A | Santa Cruz Biotechnology, Santa Cruz, CA, USA | sc-108080 |
| TRAF4 shRNA (h) Lentiviral Particles | Santa Cruz Biotechnology, Santa Cruz, CA, USA | sc-36713-V |
| siGENOME non-targeting siRNA Control Pools | [Horizon](https://horizondiscovery.com/en/gene-modulation/knockdown/sirna/products/sigenome-sirna-reagents) Discovery, [Waterbeach, United Kingdom](https://www.google.com/search?q=Waterbeach&stick=H4sIAAAAAAAAAOPgE-LSz9U3qEoqzjHOUeIEsc2Mi-OTtLSyk63084vSE_MyqxJLMvPzUDhWGamJKYWliUUlqUXFi1i5whOBjKTUxOSMHayMAOSD6DtVAAAA&sa=X&ved=2ahUKEwipn8yc0ZP0AhXQkWoFHaYMA5oQmxMoAXoECEAQAw) | D-001206-13-5 |
| siGENMONE SMARTpool TRAF4 siRNA | [Horizon](https://horizondiscovery.com/en/gene-modulation/knockdown/sirna/products/sigenome-sirna-reagents) Discovery, [Waterbeach, United Kingdom](https://www.google.com/search?q=Waterbeach&stick=H4sIAAAAAAAAAOPgE-LSz9U3qEoqzjHOUeIEsc2Mi-OTtLSyk63084vSE_MyqxJLMvPzUDhWGamJKYWliUUlqUXFi1i5whOBjKTUxOSMHayMAOSD6DtVAAAA&sa=X&ved=2ahUKEwipn8yc0ZP0AhXQkWoFHaYMA5oQmxMoAXoECEAQAw) | M-006908-01-0005 |
| lentiCRISPR v2 plasmid | Addgene, [Watertown, MA](https://www.google.com/search?q=Watertown,+Massachusetts&stick=H4sIAAAAAAAAAOPgE-LSz9U3yEsrNzQ0UOIAsUviLcq1jDLKrfST83NyUpNLMvPz9POL0hPzMqsSQZxiq4zUxJTC0sSiktSiYoWc_GSw8CJWifBEoEhJfnmejoJvYnFxYnJGaXFqSUnxDlZGAGsfOmNqAAAA&sa=X&ved=2ahUKEwiAwPunxvzzAhWaHc0KHa2yBAIQmxMoAXoECGIQAw), USA | 52961 |
| pRK5-HA-Ubiquitin-WT plasmid | Addgene, [Watertown, MA](https://www.google.com/search?q=Watertown,+Massachusetts&stick=H4sIAAAAAAAAAOPgE-LSz9U3yEsrNzQ0UOIAsUviLcq1jDLKrfST83NyUpNLMvPz9POL0hPzMqsSQZxiq4zUxJTC0sSiktSiYoWc_GSw8CJWifBEoEhJfnmejoJvYnFxYnJGaXFqSUnxDlZGAGsfOmNqAAAA&sa=X&ved=2ahUKEwiAwPunxvzzAhWaHc0KHa2yBAIQmxMoAXoECGIQAw), USA | 17608 |
| pRK5-HA-Ubiquitin-K48 plasmid | Addgene, [Watertown, MA](https://www.google.com/search?q=Watertown,+Massachusetts&stick=H4sIAAAAAAAAAOPgE-LSz9U3yEsrNzQ0UOIAsUviLcq1jDLKrfST83NyUpNLMvPz9POL0hPzMqsSQZxiq4zUxJTC0sSiktSiYoWc_GSw8CJWifBEoEhJfnmejoJvYnFxYnJGaXFqSUnxDlZGAGsfOmNqAAAA&sa=X&ved=2ahUKEwiAwPunxvzzAhWaHc0KHa2yBAIQmxMoAXoECGIQAw), USA | 17605 |
| pRK5-HA-Ubiquitin-K63 plasmid | Addgene, [Watertown, MA](https://www.google.com/search?q=Watertown,+Massachusetts&stick=H4sIAAAAAAAAAOPgE-LSz9U3yEsrNzQ0UOIAsUviLcq1jDLKrfST83NyUpNLMvPz9POL0hPzMqsSQZxiq4zUxJTC0sSiktSiYoWc_GSw8CJWifBEoEhJfnmejoJvYnFxYnJGaXFqSUnxDlZGAGsfOmNqAAAA&sa=X&ved=2ahUKEwiAwPunxvzzAhWaHc0KHa2yBAIQmxMoAXoECGIQAw), USA | 17606 |
| SMURF2 Active human protein | Sigma-Aldrich Inc. St. Louis, MO, USA. | SRP0228 |
| Recombinant human MYC tagged HER2 protein | Origene, [Rockville, MD](https://www.google.com/search?q=Rockville,+Maryland&stick=H4sIAAAAAAAAAOPgE-LSz9U3qEpPMjUpV-IAsZMqkpK0tLKTrfTzi9IT8zKrEksy8_NQOFYZqYkphaWJRSWpRcWLWIWD8pOzyzJzclJ1FHwTiypzEvNSdrAyAgAR6wjwXQAAAA&sa=X&ved=2ahUKEwjD-NqaxfzzAhWRZM0KHTjhDWEQmxMoAXoECDcQAw), USA | TP312583 |
| Recombinant human GST tagged TRAF4 protein | Origene, [Rockville, MD](https://www.google.com/search?q=Rockville,+Maryland&stick=H4sIAAAAAAAAAOPgE-LSz9U3qEpPMjUpV-IAsZMqkpK0tLKTrfTzi9IT8zKrEksy8_NQOFYZqYkphaWJRSWpRcWLWIWD8pOzyzJzclJ1FHwTiypzEvNSdrAyAgAR6wjwXQAAAA&sa=X&ved=2ahUKEwjD-NqaxfzzAhWRZM0KHTjhDWEQmxMoAXoECDcQAw), USA | TP761293 |
| Recombinant human FLAG tagged SMURF2 protein | Creativebiomart, Shirley, NY, USA | SMURF2-468H |

**Supplementary Table 2. List of primers.**

| HER2 Q-PCR Primer | F: 5’-GGAAGTACACGATGCGGAGACT-3’ |
| --- | --- |
|  | R: 5’-ACCTTCCTCAGCTCCGTCTCTT-3’ |
| TRAF4 Q-PCR Primer | F: 5’-GCCGTGATCTACCTGCACACTT-3’ |
|  | R: 5’-CTCACAGTAGACACTCTCCTGG-3’ |
| SMURF2 Q-PCR Primer | F: 5’-TCCTCGGCTGTCTGCTAACTTG-3’ |
|  | R: 5’-CAGGCATTCTGTGTCATCAGGAC-3’ |
| GAPDH Q-PCR Primer | F: 5’-GTCTCCTCTGACTTCAACAGCG-3’ |
|  | R: 5’-ACCACCCTGTTGCTGTAGCCAA-3’ |
| TRAF4 CRISPR-sgRNA-1 | 5'-GTACTTCCAGCTCCGGGTCT-3' |
| TRAF4 CRISPR-sgRNA-2 | 5'-TGTGGATGCAGCGGATAGGC-3' |
| TRAF4 KO sequencing primer | F: 5'-CCCGCAAGAAGTGTTCCCTC-3' |
|  | R: 5'-CTGTCACCTACCCTGCAACCC-3' |
| TRAF4 C80A | F: 5'-CGACGGCTGCTGGCCCCACTGTGC-3’ |
|  | R: 5'-CTTGGGCTTCTCCAGGAACTTGTA-3’ |
| SMURF2 C716A | F: 5'-GCCCACACTGCCTTCAATCGAATA-3’ |
|  | R: 5'-TTTCGGCAGGTTGTTAGTGCAGGC-3’ |
| HER2-ΔECD | F: 5’-GCCGAGCAGAGAGCCAGCCCTCTG-3’ |
|  | R: 5’- GCTACCGCCTCCACCGGCGTAGTC-3’ |
| HER2- ΔICD | F: 5’-TACCTGGGTCTGGACGTGCCAGTG-3’ |
|  | R: 5’-CTTCCGGATCTTCTGCTGCCGTCGCTT-3’ |

**Supplementary Table 3. List of target sequences for siRNA or shRNA mediated gene knockdown.**

| Products | Company | Sequences (5′ → 3′) |
| --- | --- | --- |
| siGENMONE SMARTpool TRAF4 siRNA | [Horizon](https://horizondiscovery.com/en/gene-modulation/knockdown/sirna/products/sigenome-sirna-reagents) Discovery, [Waterbeach, United Kingdom](https://www.google.com/search?q=Waterbeach&stick=H4sIAAAAAAAAAOPgE-LSz9U3qEoqzjHOUeIEsc2Mi-OTtLSyk63084vSE_MyqxJLMvPzUDhWGamJKYWliUUlqUXFi1i5whOBjKTUxOSMHayMAOSD6DtVAAAA&sa=X&ved=2ahUKEwipn8yc0ZP0AhXQkWoFHaYMA5oQmxMoAXoECEAQAw) | 1. GAAACUAUGUGCGGGAUGA |
|  |  | 2. UGAUCUACCUGCACACUUG |
|  |  | 3. GGCCACCGUUUCUGCGAUA |
|  |  | 4. CAUCCGUGCUGCUGUUGAA |
| siGENMONE SMARTpool SMURF2 siRNA | [Horizon](https://horizondiscovery.com/en/gene-modulation/knockdown/sirna/products/sigenome-sirna-reagents) Discovery, [Waterbeach, United Kingdom](https://www.google.com/search?q=Waterbeach&stick=H4sIAAAAAAAAAOPgE-LSz9U3qEoqzjHOUeIEsc2Mi-OTtLSyk63084vSE_MyqxJLMvPzUDhWGamJKYWliUUlqUXFi1i5whOBjKTUxOSMHayMAOSD6DtVAAAA&sa=X&ved=2ahUKEwipn8yc0ZP0AhXQkWoFHaYMA5oQmxMoAXoECEAQAw) | 1. GAUGAGAACACUCCAAUUA |
|  |  | 2. GACCAUACCUUCUGUGUUG |
|  |  | 3. CAAAGUGGAAUCAGCAUUA |
|  |  | 4. GAACAACACAAUUUACAGA |
| TRAF4 shRNA (h) | Santa Cruz Biotechnology, Santa Cruz, CA, USA | Sense1: UCAGUGAAGGAGUCUUCAA |
|  |  | Antisense1: UUGAAGACUCCUUCACUGA |
|  |  | Sense2: CUGGACUAUGCCAAGAUCU |
|  |  | Antisense 2: AGAUCUUGGCAUAGUCCAG |
|  |  | Sense3: GGCUUUGGUUAUCCCAAGU |
|  |  | Antisense 3: ACUUGGGAUAACCAAAGCC |

SMARTpool: A mixture of 4 siRNA provided as a single reagent.

TRAF4 shRNA (h): a pool of 3 different shRNA plasmids.

**Supplementary Table 4. Colocalization coefficients (Pearson product-moment correlation coefficient (PCC)) of TRAF4, SMURF2 and HER2 in HER2+ breast cancer cells.**

| Overlap Coefficient | HCC1954 | BT474 | MDA-MB-453 | HCC1419 |
| --- | --- | --- | --- | --- |
| TRAF4/SMURF2 | 0.99 | 0.94 | 0.93 | 0.88 |
| TRAF4/HER2 | 0.96 | 0.94 | 0.98 | 0.89 |
| SMURF2/HER2 | 0.97 | 0.94 | 0.92 | 0.95 |
